# Inbreeding Depression in Genotypically Matched Diploid and Tetraploid Maize

**DOI:** 10.3389/fgene.2020.564928

**Published:** 2020-11-30

**Authors:** Hong Yao, Sanvesh Srivastava, Nathan Swyers, Fangpu Han, Rebecca W. Doerge, James A. Birchler

**Affiliations:** ^1^Division of Biological Sciences, University of Missouri, Columbia, MO, United States; ^2^Department of Statistics and Actuarial Science, University of Iowa, Iowa City, IA, United States; ^3^Department of Statistics, Carnegie Mellon University, Pittsburgh, PA, United States

**Keywords:** maize, tetraploid, heterosis, progressive heterosis, inbreeding depression

## Abstract

The genetic and molecular basis of heterosis has long been studied but without a consensus about mechanism. The opposite effect, inbreeding depression, results from repeated self-pollination and leads to a reduction in vigor. A popular explanation for this reaction is the homozygosis of recessive, slightly deleterious alleles upon inbreeding. However, extensive studies in alfalfa indicated that inbreeding between diploids and autotetraploids was similar despite the fact that homozygosis of alleles would be dramatically different. The availability of tetraploid lines of maize generated directly from various inbred lines provided the opportunity to examine this issue in detail in perfectly matched diploid and tetraploid hybrids and their parallel inbreeding regimes. Identical hybrids at the diploid and tetraploid levels were inbred in triplicate for seven generations. At the conclusion of this regime, F1 hybrids and selected representative generations (S1, S3, S5, S7) were characterized phenotypically in randomized blocks during the same field conditions. Quantitative measures of the multiple generations of inbreeding provided little evidence for a distinction in the decline of vigor between the diploids and the tetraploids. The results suggest that the homozygosis of completely recessive, slightly deleterious alleles is an inadequate hypothesis to explain inbreeding depression in general.

## Introduction

Heterosis refers to the phenomenon that hybrid progeny of inbred parents will exceed the performance of the better parent (Shull, 1908; Bruce, 1910; Chen, 2013). It has been capitalized upon by plant breeders for decades to enhance yields, but its genetic and molecular basis has escaped understanding. A popular concept to explain heterosis has been that slightly deleterious homozygous mutations that differ in the parents are complemented in the hybrid (Jones, 1917). To the degree that deleterious mutations are present and homozygous in the different inbreds, this complementation will certainly occur. However, modern inbreds might have been purged of the obviously deleterious mutations from heterotic groups but still exhibit a robust heterotic effect when crossing inbreds from the opposite groups (Duvick, 1999). This concept is derived from work on heterosis in ostensibly diploid species.

Indeed, the behavior of heterosis in polyploids is not prima facie explicable on this hypothesis (East, 1936; Briggle, 1963; Li et al., 2008). First, the phenomenon of progressive heterosis has been documented in several tetraploid plants including alfalfa, potato, and maize (Levings et al., 1967; Dunbier and Bingham, 1975; Mok and Peloquin, 1975; Bingham, 1980; Chase, 1980; Groose et al., 1989; Sockness and Dudley, 1989a,b; Bingham et al., 1994; Riddle et al., 2010; Washburn et al., 2019). This phenomenon is that double-cross hybrids resulting from a cross of two different single cross hybrids exhibit a further increase in heterosis. Symbolically, this can be illustrated in that an ABCD hybrid shows greater heterosis than AABB or CCDD. In contrast, double-cross hybrids at the diploid level do not routinely show this response (Washburn et al., 2019). In order for complementation to explain progressive heterosis, the AAAA homozygous lines would need to have different detrimental alleles than BBBB but some in common. At the same time CCCC would need to have a different set than DDDD but some different alleles in common so that AABB and CCDD would, respectively, still be homozygous for different sets to complement in the double-cross hybrid. All these conditions would need to be met in order not to reconstitute a homozygous state for detrimental recessives (Washburn et al., 2019).

A second observation that is not explained by the complementation concept is that heterosis is different in reciprocal triploid hybrids despite being quite similar in diploid hybrids (i.e., AAB ≠ BBA but AB = BA) (Yao et al., 2013; Tan et al., 2016). If recessive detrimentals were the sole basis of heterosis, then AAB and BBA should be similar. The fact that they are routinely distinct indicates that there is a dosage component to heterosis.

The third observation of note from polyploidy heterosis is that inbreeding depression curves of matched diploid and tetraploid genotypes are quite similar despite a very different predicted rate of homozygosis. This effect has been studied in alfalfa, wheatgrass, and to a lesser degree in maize (Alexander and Sonnemaker, 1961; Demarly, 1963; Busbice and Wilsie, 1966; Dewey, 1966, 1969; Rice and Dudley, 1974; Chase, 1980; Gallais, 1984; Li and Brummer, 2009). Indeed, in the early days of alfalfa breeding, this parallel led to great confusion (Williams, 1931; Tysdal et al., 1942). For a single gene in a diploid, self-pollination of a heterozygote would produce a quarter of the progeny that are homozygous for either of the two alleles present (Charlesworth and Willis, 2009). However, in a autotetraploid duplex hybrid (AABB), self-pollination would produce only 1/36 of the progeny that would be homozygous for either allele for genes near the centromeres. This tetraploid estimate is subject to many caveats such as recombination between a locus being followed and the centromere as well as the mode of segregation of the four homologous chromosomes during meiosis I. Nevertheless, during an inbreeding regime the rate of homozygosis in an autotetraploid would be predicted to be considerably slower than in a diploid (Welch, 1962; Levings and Alexander, 1966; Doyle, 1979, 1986; Birchler, 2012). The fact that the decline in vigor between diploids and autotetraploids is quite similar suggests an explanation for heterosis and inbreeding depression needs further explanation than complementation of recessive mutations (Washburn et al., 2019).

The subject of the present study was a test of the rate of inbreeding depression between diploids and autotetraploids that were directly derived from the diploid inbreds and thus of exactly the same genotype. Previous studies in maize were of a preliminary nature and the tetraploids might have some differences with the diploids with which they were compared. The results of the present study reveal that indeed there is a very similar rate of inbreeding depression upon selfing of comparable genotypes of the starting hybrid materials at the diploid and tetraploid levels.

## Materials and Methods

The experiments to investigate inbreeding depression rates in diploid and tetraploid maize lines were conducted in 2009 (Supplementary Material 1) and 2008 (Supplementary Material 2) in Columbia, Missouri. The four diploid and tetraploid parental inbred maize lines A188 (2x, 4x), Oh43 (2x, 4x), B73 (2x, 4x), and W22 (2x, 4x) were used in this study (Kato and Birchler, 2006; Riddle et al., 2006; Riddle and Birchler, 2008). The following F1 hybrids from these parental lines were grown: Oh43/A188 (2x), A188/Oh43 (2x), W22/B73 (2x), B73/W22 (2x), Oh43/W22 (2x), W22/Oh43 (2x), W22/A188 (2x), A188/W22 (2x), B73/A188 (2x), A188/B73 (2x), B73/Oh43 (2x), Oh43/B73 (2x), A188/Oh43 (2x) × B73/W22 (2x), B73/W22 (2x) × A188/Oh43 (2x), W22/B73 (4x), A188/Oh43 (4x), Oh43/A188/W22/B73 (4x).

Each F1 hybrid line was previously self-mated for seven generations and progenies from generations 1, 3, 5, and 7 (named S1, S3, S5, and S7) were used for data collection. Genetic segregation occurred after the first generation of the self-mating population in this experiment. Kernels from three different S2 ears (resulting from self-mating S1 plants) were used to produce the S3–S7 lines, to account for the genetic diversity among the S1 plants derived from the same F1 hybrid. Thus, there were three selfing lineages for each genotype.

The experiments were based on a randomized complete block design. The maize lines from all ploidy levels, genotypes, and generations were planted in three fields. Each maize line was grown in each of the three fields (blocks) with border rows of unrelated maize. All genotypes were randomized within the blocks with intermixing of the diploid and tetraploid samples. Soil type was “Leonard silt loam” or “Mexico silt loam.” Twenty seeds of the respective maize lines were planted per row with 22.96 cm spacing between plants in a row, and, whenever possible, data from at most 12 plants were collected. The planting dates of the three blocks in 2009 were May 21, June 1, and June 14 at the University of Missouri Genetic Farm near Columbia, Missouri.

Data on the following phenotypes were collected (the names in parentheses denote the names used in the analysis for the corresponding phenotype): (1) the number of days to anther emergence after planting (flowering time); (2) the number of days to silk emergence after planting (silk emergence time); (3) the ear length of the maize plant (ear length); (4) the tassel branch number (tassel branch number); (5) the height of the plant to the growing tip at the fourth week (4th week height); (6) the height of the plant to the growing tip at the sixth week (6th week height); (7) the height of the adult plant to the top of the tassel (adult plant height); (8) the length of the fifth leaf from the top of the adult plant (length of the 5th leaf from the top), and (9) the width of the fifth leaf from the top of the adult plant (width of the 5th leaf from the top).

The experiments were conducted to investigate the following biological questions about inbreeding depression rates:

1. Is the inbreeding depression different between diploid and tetraploid lines with the same genetic constitution?
2. Is the inbreeding depression different between lines with different genetic constitution but the same ploidy?
3. Does inbreeding depression occur in all the measured phenotypes?
4. Is there inbreeding depression in every diploid and tetraploid genotype?
5. How is the inbreeding depression rate affected by ploidy, genetic constitution, and the interaction between ploidy and genetic constitution?
6. Are there any parental effects on inbreeding depression rate?
7. Are the S7 lines different from their corresponding progenitor inbred lines?

### Statistical Analyses

We have summarized the data for every field and phenotype by averaging over the biological replicates. Separately, the summarized data from the different fields and years are analyzed. The generations F0, S1, S3, S5, and S7 are labeled as 0, 1, 3, 5, and 7, respectively. Let *y* be the observed phenotypic value in generation *gen* ∈ {0, 1, 3, 5, 7} and *ploidy* be an indicator variable that is 0 and 1 for diploid and tetraploid plants, respectively. If ϵ and *geno* are the idiosyncratic error in measuring the phenotype and dummy variable denoting the genotype of the plant, then we model *y* using two different models depending on the question:

$$y=\beta_{0}+\beta_{1}\,g\,e\,n+\beta_{2}\,p\,l\,o\,i\,d\,y+\beta_{3}\,\left(g\,e\,n\times p\,l\,o\,i\,d\,y\right)+\varepsilon ,$$

$$y=\beta_{0}+\beta_{1}\,g\,e\,n+\beta_{2}\,g\,e\,n\,o+\beta_{3}\,\left(g\,e\,n\times g\,e\,n\,o\right)+\varepsilon ,$$

where *β*_1_, *β*_2_, *β*_3_ are the regression coefficients; *β*_0_ is the intercept; and the former and latter models account for the interaction of generation with ploidy and generation with genotype, respectively. If inbreeding depression is present in a phenotype, then *β*_1_ is negative. Our hypotheses tests are performed under the additional assumption that ϵ is Gaussian with mean 0. In both models, our questions are answered by testing the null hypothesis *β*_3_ = 0; see Supplementary Materials 1–4 for greater details.

### Segregation Patterns

In order to test whether the chromosome segregation from the tetraploid hybrids followed tetrasomic or disomic patterns, progeny from self-pollinated ears of a W22/B73 hybrid were screened for the distribution of W22 or B73 chromosomes using fluorescence *in situ* hybridization (FISH). The following is a description of the distinctions between the two inbred lines for chromosomes that differ in cytological features (see also Albert et al., 2010).

Chromosome 1: Chromosome 1 can be distinguished between W22 and B73 by the strong TAG probe signal on the long arm of W22 chromosome 1 as compared to B73 chromosome 1 as well as the 4-12-1 probe signal on the short arm of W22 chromosome 1.

Chromosome 2: Chromosome 2 can be distinguished by the presence of the 5S gene cluster and then the strong CentC probe signal on W22 chromosome 2 contrasted by the very weak CentC signal on B73 chromosome 2 distinguishing the two genotypes from each other.

Chromosome 4: Chromosome 4 can be distinguished by the presence of a unique centromeric sequence (Cent4) and then the distinctive knob highlighted by DAPI stain on W22 chromosome 4 as well as the strong microsatellite TAG probe signal on the short arm of B73 chromosome 4 allowing the two genotypes to be determined.

Chromosome 5: B73 chromosome 5 displays a much stronger 4-12-1 probe signal on its short arms than its W22 counterpart. There also is a much stronger knob signal revealed by DAPI staining on W22 chromosome 5 than B73 chromosome 5.

Chromosome 8: W22 chromosome 8 has a much stronger CentC probe signal than B73 chromosome 8. Chromosome 8 can be determined by a consistent subtelomeric signal on the long arm.

Chromosome 9: Chromosome 9 has a consistent terminal knob on the short arm, but W22 chromosome 9 has a much stronger CentC probe signal than B73 chromosome 9.

Chromosome 10: Chromosome 10 is the shortest chromosome and can be distinguished from the other chromosomes by the absence of any consistent markers. W22 chromosome 10 has a much stronger CentC probe signal than B73 chromosome 10.

### Fluorescence *in situ* Hybridization (FISH)

FISH was conducted as described (Kato et al., 2004). The set of probes used in this experiment is as follows: The CentC probe was labeled green and identifies a centromeric repeat. The 4-12-1 probe is labeled green and consists of a subtelomeric repeat. The NOR probe is labeled green and denotes the nucleolar organizing region. The TAG satellite probe is labeled red. The Cent4 probe is labeled red and shows a centromeric repeat specific to chromosome 4. Finally, DAPI is used to stain heterochromatin. Not all of the chromosome pairs could be accurately distinguished between W22 and B73, so those were not included in the analysis. Chromosomes 1, 2, 4, 5, 8, 9, and 10 were used because parental genotypes were able to be distinguished in the tetraploid. Green probes are labeled using AlexaFluor dUTPs, and red probes are labeled using TexasRed dCTPs.

## Results

As detailed in Supplementary Materials 1–4, the null hypothesis that the rate of inbreeding depression between diploids and tetraploids is different could be rejected in the vast majority of comparisons of genotypes in the four generations of selfing progression that were analyzed (S1, S3, S5, S7). The different genotypes, however, could be distinguished from each other at the diploid level, but some comparisons at the tetraploid level were not significant (see Supplementary Materials 1–3. Supplementary Material 4 contains the probabilities in tabular form.). All of the measured phenotypic characteristics exhibited inbreeding depression and the depression was observed at both ploidy levels examined. There was not sufficient data to make a generalization of whether the S7 was different from the corresponding inbred lines.

**FIGURE 1:**
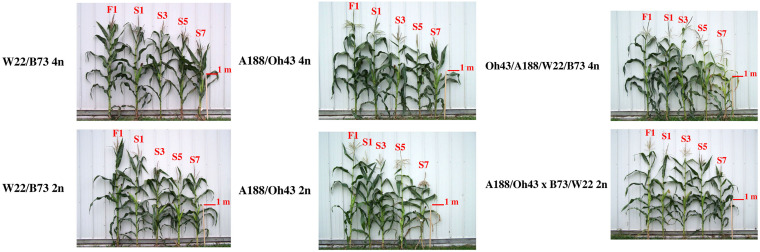
Comparison of inbreeding of a W22/B73 hybrid for seven generations at the tetraploid and diploid levels. (Top) Examples of plants from the inbred generations from the F1 tetraploid to the seventh selfed generation. (Bottom) Examples from the diploid progression. One meter stick provides scale.

**FIGURE 2:**
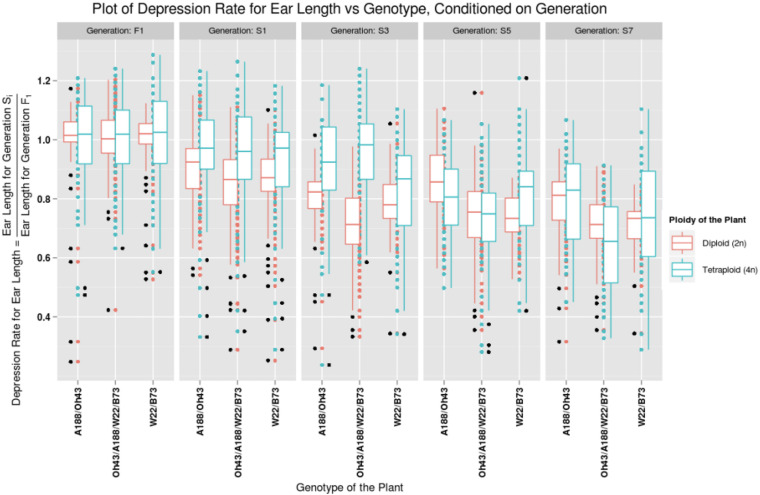
Plot of depression rate for ear length vs. the genotype of the plant, conditioned on generations F1, S1, S3, S5, and S7. The data for diploid plants are graphed in red and the tetraploid plants are graphed in blue. The patterns for a particular ploidy can be observed by looking at the box plots of the corresponding color. The generation varies from F1 to S7 column-wise.

Figure 1 illustrates one comparison of adult plant height from the F1 to the S7 for the W22/B73 diploid and tetraploid, the A188/Oh43 diploid and tetraploid, and the double-cross hybrid at the diploid and tetraploid levels. Figure 2 shows the matched diploid and tetraploid measurements for the three comparisons from the F1 generation to the S7 for ear length. The plots of depression rate for the other characteristics measured are in Supplementary Materials 1–3.

**FIGURE 3:**
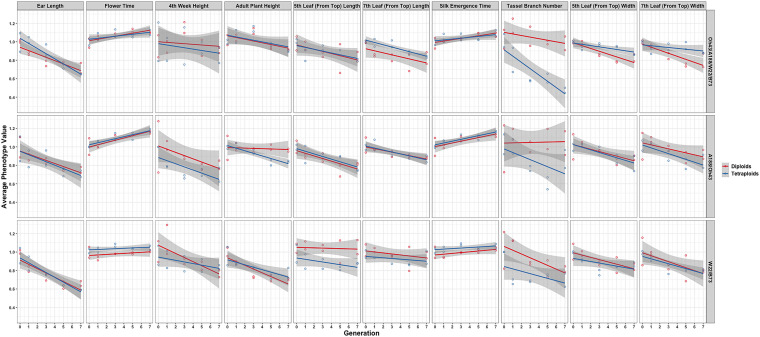
Visualization of the interaction between ploidy and genotype in the 2008 data. Generation (*x*-axis) vs. averaged phenotypic values across biological replicates (*y*-axis) conditioned on the genetic constitution (rows) and trait (columns). Two linear regression lines are superimposed on every panel and their color indicates the diploid (red) and tetraploid plants (blue). The gray-colored band on a regression indicates a 95% confidence interval for the whole line.

**FIGURE 4:**
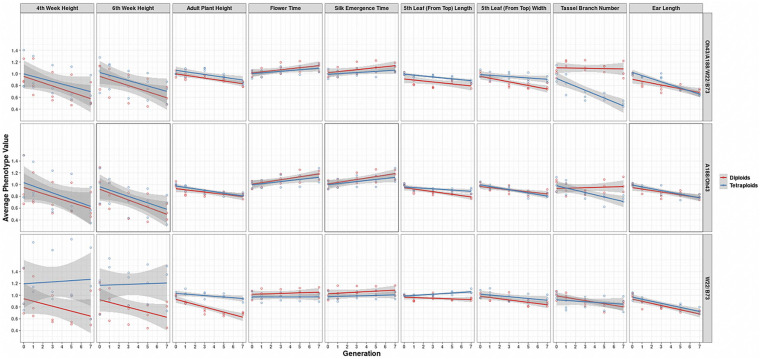
Visualization of the interaction between ploidy and genotype in the 2009 data. Generation (*x*-axis) vs. averaged phenotypic values across biological replicates (*y*-axis) conditioned on the genetic constitution (rows) and trait (columns). Two linear regression lines are superimposed on every panel and their color indicates the diploid (red) and tetraploid plants (blue). The gray-colored band on a regression indicates the 95% confidence band for the whole line.

**FIGURE 5:**
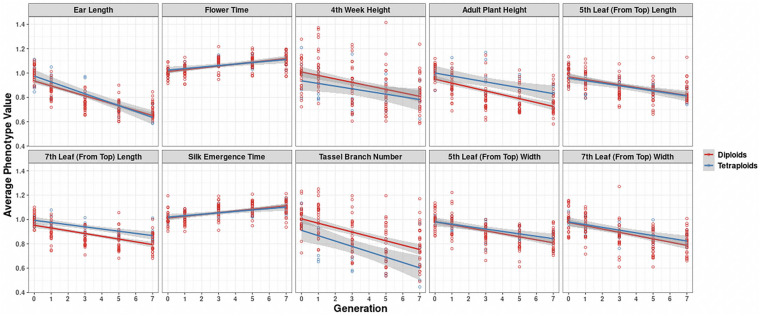
Visualization of inbreeding depression for the 10 phenotypes in the 2008 data conditioned on ploidy. Generation (*x*-axis) vs. averaged phenotypic values across biological replicates (*y*-axis) for the 10 traits (panels). Two linear regression lines are superimposed on every panel, and their color indicates the diploid (red) and tetraploid plants (blue). The gray-colored band on a regression indicates the 95% confidence band for the whole line.

**FIGURE 6:**
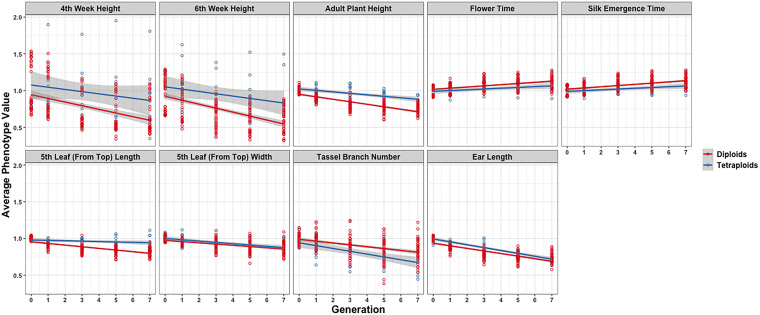
Visualization of inbreeding depression for the nine phenotypes in the 2009 data conditioned on ploidy. Generation (*x*-axis) vs. averaged phenotypic values across biological replicates (*y*-axis) for the 10 traits (panels). Two linear regression lines are superimposed on every panel and their color indicates the diploid (red) and tetraploid plants (blue). The gray-colored band on a regression indicates the 95% confidence band for the whole line.

In further analysis, the averaged phenotypic values across biological replicates were plotted across generations for the various traits examined [Figure 3 (2008 data) and Figure 4 (2009 data); Supplementary Materials 3, 4] separated into the three genotypes. The linear regression lines for the two ploidies are not significantly different for most traits and genotypes between diploid and tetraploid. A potential exception is the trait of the tassel branch number. However, the slope of the tetraploid is steeper than that of the diploid in those cases in which they differ suggesting a stronger effect of inbreeding in the tetraploids for this trait. Figure 5 (2008 data) and Figure 6 (2009 data) plot the average phenotypic values vs. generation for the traits measured in this case using data for all genotypes and all fields together. Overall, there is not a clear distinction in the diploid and tetraploid comparison.

**FIGURE 7:**
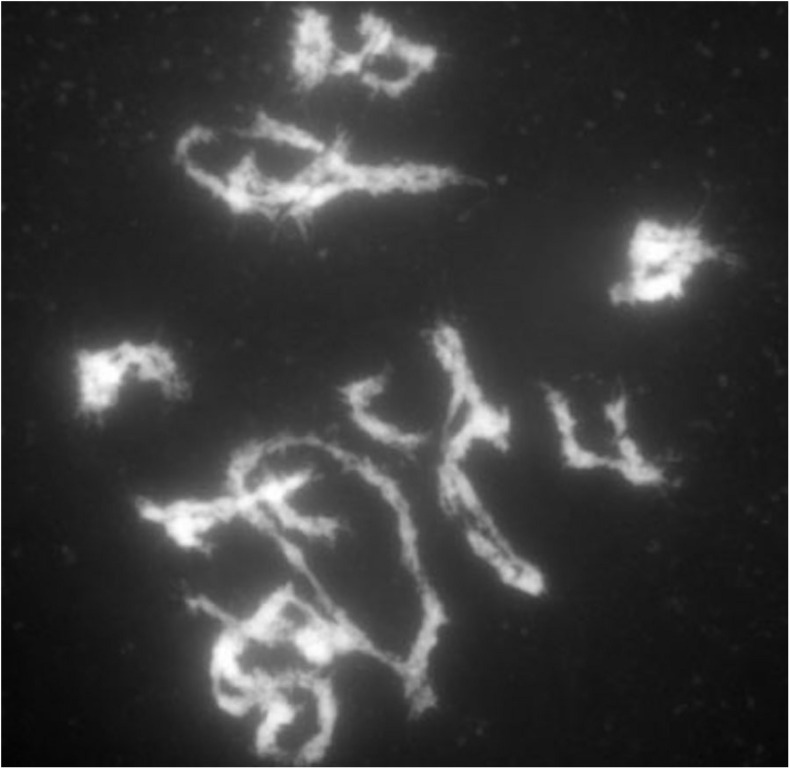
Late prophase of meiosis I in a tetraploid B73. Carmine stain of late prophase of a tetraploid B73 plant. Note the array of bivalents and multivalents.

Because there is not a clear distinction of the inbreeding depression plots between the diploids and tetraploids, we considered the possibility that the chromosomal segregation in the tetraploid might not be as expected. The unlikely scenario that only unlike homologues would pair with each other and that like homologues would proceed to the same pole from such pairing might produce the observed inbreeding curves because that scenario would mimic diploid segregation, although this scenario would still predict a slower inbreeding progression than in diploids, being 1/8 vs. 1/2 at any one locus. To examine this possibility, early, and late meiosis I samples of selected homozygous or heterozygous tetraploid genotypes were examined (Figure 7). The patterns of pairing observed indicated the typical array of tetraploid pairing involving bivalents, trivalents, and quadrivalents.

To examine this issue further, chromosomal karyotypes were performed on selfed progeny of tetraploid W22/B73 hybrid plants. In this comparison, markers on chromosomes 1, 2, 4, 5, 8, 9, and 10 could be distinguished. Tetraploid maize plants were analyzed using FISH. A cocktail of probes was used to identify the separate chromosomes in the plants as well as the genetic background, W22 or B73, of those chromosomes.

Using the noted chromosomal features, several tetraploid plants were karyotyped. There were 18 plants that were able to be examined. Using the karyotypes, chromosome totals were obtained representing those from the W22 genotype, those from the B73 genotype as well as both genotypes together. A total of 372 chromosomes were assigned to the two genotypes: 193 W22 chromosomes and 179 B73 chromosomes. Chromosome sets that were not complete or could not be assigned to either genotype were excluded from the data set. Chromosome set categories were assigned based on the distribution of W22 chromosomes and B73 chromosomes for each tetraploid chromosome number. The sets were as follows with W22 being the first number and B73 being the second: 4:0, 3:1, 2:2, 1:3, and 0:4. These sets were tallied, and a chi-square test was performed on the observed numbers of each category to test if they followed a distribution of 1:8:18:8:1 predicted from that random joining of gametes in an autotetraploid using the centromere as the marker. There were a total of 92 tallied individual distributions. The observed distributions for the five separate categories were 4:0 = 6, 3:1 = 20, 2:2 = 41, 1:3 = 23, and 0:4 = 2. The chi-square for these data is 5.60 with 4° of freedom and a *p* > 0.10. Thus, the deviation from the predicted segregation for an autotetraploid is not significant.

## Discussion

In this study, the pattern of inbreeding depression was compared at the diploid and tetraploid levels in matched genotypes starting at the F1 hybrid stage. The connection among allelic constitutions, genic interactions in the genome, and the phenotype is not understood, including how inbreeding across the genome intersects with these considerations. Nevertheless, there is not a clear distinction that inbreeding depression is slower for tetraploids than diploids. Indeed, for some characteristics, there is an accelerated depression. Various studies of heterosis in diploids have indicated that different characteristics are not necessarily correlated with regard to the magnitude of heterosis (Flint-Garcia et al., 2009; Yao et al., 2013). This relationship is apparent in these data as well. Nevertheless, the overall trend is that there is not an obvious difference between the inbreeding patterns at the two ploidy levels.

On the assumption that inbreeding results from the homozygosis of slightly deleterious recessive alleles, the results are not consistent with this concept. The homozygosis of alleles in an autotetraploid depends on the position of the gene on the chromosome because recombination between the gene in question and the centromere will allow double reduction to occur, i.e., the entry of the same allele from two homologues to a meiotic end product to produce a homozygous gamete from a heterozygous parent (Levings and Alexander, 1966). However, even for genes near the tips of chromosome arms, homozygosis would not be predicted at the same rate as in a diploid.

The behavior of chromosomes in meiosis I in a representative homozygous and heterozygous genotype was found to exhibit the complicated pattern of pairing typical of autotetraploids (Randolph, 1935) with chromosomes synapsed in pairs but switching pairing partners along the length of the chromosome. The pairing configurations at the end of meiosis I showed pairs, trivalents, and quadrivalents as would be predicted from the pairing associations at the pachytene stage. Distinguishing chromosomal features between genotypes was documented in progeny of a selfed heterozygote and revealed no significant deviation from the expectations of tetraploid frequencies. Thus, there is no reason to suspect from the chromosome behavior that segregation is unusual for the autotetraploids in this study.

The rate of recombination in diploid and tetraploids might potentially impact the rate of inbreeding depression, although there is little known about this issue. The data that are available suggest that the recombination frequency is higher in autotetraploids (Pecinka et al., 2011; Wang and Luo, 2012), but how this would intersect with chromosomal segregation and double reduction to affect homozygosis is not known.

Another factor that could impact the inbreeding curve is that tetraploids will produce many gametes that are heterozygous. There is the potential that heterozygous pollen tubes would grow faster than homozygous ones for certain loci, in other words, exhibit heterosis. Chase (1980) claimed evidence for heterosis in diploid gametophytes. Therefore, if indeed this were the case, the preferential success of heterozygous gametophytes would only be predicted to slow the rate of homozygosis even more because the opportunity to produce homozygous zygotes would be reduced. However, the data from chromosomal feature segregation suggest that there is not a greater number of heterozygous gametes to a measurable degree.

Given that chromosome behavior and heterotic gametophytes favor a slowed progression to homozygosis, what could account for the observed results? Busbice and Wilsie (1966) suggested from work in alfalfa with similar results that a shift in allelic dosage might account for the related inbreeding curves. Indeed, changing allelic dosage is more similar between diploids and tetraploids than homozygosis (Birchler, 2012). As noted earlier, heterosis in triploid hybrids shows evidence of a dosage component and perhaps those results and the ones presented here reflect a related mechanism. Many quantitative traits exhibit semidominance or dosage-sensitive effects (Birchler and Veitia, 2012; Birchler et al., 2016), and this fact raises the possibility that the control of heterosis operates at the level of regulatory interactions (Wang et al., 2015, 2017). Further work is required to understand heterosis in detail, of course, but determining its behavior under as many circumstances as possible will establish the evidence that needs to be explained by a comprehensive framework.

## Data Availability Statement

All datasets presented in this study are included in the article/Supplementary Material.

## Author Contributions

HY conducted the biological experiments. SS performed the statistical analysis. NS performed the segregation analysis. FH performed the meiosis analysis. RD supervised the statistical analysis. JB designed the experiments and wrote the manuscript. All authors contributed to the article and approved the submitted version.

## Conflict of Interest

The authors declare that the research was conducted in the absence of any commercial or financial relationships that could be construed as a potential conflict of interest.
