# Supplementary material for: Inbreeding Depression in Genotypically Matched Diploid and Tetraploid Maize
Source: Front Genet. 2020 Nov 30;11:564928. doi: 10.3389/fgene.2020.564928 (PMC7734256; doi:10.3389/fgene.2020.564928)
Supplement: Supplementary file 1 [file Data_Sheet_1.PDF]

SUPPLEMENTARY MATERIAL #1

1. INTRODUCTION

The experiments to investigate inbreeding depression rates in diploid and tetraploid maize lines were conducted during 2008 and 2009 in Columbia, Missouri. The statistical analysis in this report is for the 2009 data.

The four diploid and tetraploid parental inbred maize lines A188 (2x, 4x), Oh43 (2x, 4x), B73 (2x, 4x) and W22 (2x, 4x) were used in this study. The following F1 hybrids from these parental lines were grown:
Oh43/A188 (2x), A188/Oh43 (2x), W22/B73 (2x), B73/W22 (2x), Oh43/W22 (2x), W22/Oh43 (2x), W22/A188 (2x), A188/W22 (2x), B73/A188 (2x), A188/B73 (2x), B73/Oh43 (2x), Oh43/B73 (2x), A188/Oh43 (2x) x B73/W22 (2x), B73/W22 (2x) x A188/Oh43 (2x), W22/B73 (4x), A188/Oh43 (4x), Oh43/A188/W22/B73 (4x).

Each F1 hybrid line was self mated for seven generations and progenies from generations 1, 3, 5, and 7 (named as S1, S3, S5, and S7) were used for data collection. Genetic segregation occurred after the first generation of the self mating population in this experiment. Kernels from three different S2 ears (resulting from self mating S1 plants) were used to produce the S3 to S7 lines, to account for the genetic diversity among the S1 plants derived from the same F1 hybrid. Thus, there were three selfing lineages for each genotype.

The experiments conducted in 2009 were based on a randomized complete block design. The maize lines from all ploidy, genotypes and generations were planted in three fields. Each maize line was grown in each of the three fields (blocks). Twenty seeds of maize lines were planted per row and, whenever possible, data from at most twelve plants were collected. The planting dates for the three blocks are as follows:

**Year 2009**
**Block 1** – May 21st
**Block 2** – June 1st
**Block 3** – June 14th

Data on the following phenotypes were collected (the names in parentheses denote the names used in the analysis for the corresponding phenotype):

1. The number of days to anther emergence after planting. (flowering time)
2. The number of days to silk emergence after planting. (silk emergence time)
3. The ear length of the maize plant. (ear length)
4. The tassel branch number. (tassel branch number)
5. The height of the plant at 4th week. (4th week height)
6. The height of the plant at 6th week. (6th week height)
7. The height of the adult plant. (adult plant height)
8. The length of the 5th leaf from the top. (length of the 5th leaf from the top)
9. The width of the 5th leaf from the top. (width of the 5th leaf from the top)

The experiments were conducted to investigate the following biological questions about inbreeding depression rates:

1. Is the inbreeding depression rate different between diploid and tetraploid lines with the same genetic constitution?
2. Is the inbreeding depression rate different between lines with different genetic constitution but the same ploidy?
3. Does inbreeding depression occur in all the measured phenotypes?
4. Is there depression in every diploid and tetraploid genotype?
5. How is the inbreeding depression rate affected by ploidy, genetic constitution and the interaction between ploidy and genetic constitution?
6. Are there any parental effects on inbreeding depression rate?
7. Are the S7 lines different from their corresponding progenitor inbred lines?

The inbreeding depression rate for a phenotypic datum corresponding to the mth replicate from

the lth field, in generation i, with genotype j, and ploidy k is defined as:

$$\text{Inbreeding Depression Rate}_{ijklm} = \frac{\text{Phenotype}_{ijklm}}{\sum_{m=1}^{12}\sum_{l=1}^{3}\text{Phenotype}_{1jklm}/36} \quad (1)$$

For example, the depression rate of ear length (phenotype) for any observation in Si generation of A188/Oh43 (2x) (genotype and ploidy) is defined/calculated as:

$$= \frac{\text{Observed Ear Length in } S_i \text{ from A188/Oh43 (2n)}}{\text{Mean of Ear Length in } F_1 \text{ from A188/Oh43 (2n)}}$$

The rest of this document is structured as follows: there is an Exploratory Data Analysis section that details the data visualization methods and plots for inbreeding depression rates for all the phenotypes. After that there is a Methodology section that details the statistical methods used in the analysis. After the Methodology section, there is a Results section discussing the result for each biological question in the analysis. An Appendix list the SAS codes used in the analysis.

2. Exploratory Data Analysis

Figure 2.1 shows the density plot for all the phenotypes grouped according to field. A density plot of a variable is an estimate of its probability density function based on the observed data under the assumption of random sampling from the population. In this particular case, the estimate of the unobservable probability density of a phenotype is plotted for the three different fields separately. This figure allows us to check for the heterogeneity of data collected from different fields. The level of heterogeneity will be used to decide whether to plot the inbreeding depression rates of phenotypes based on different fields or to pool the data from the fields.

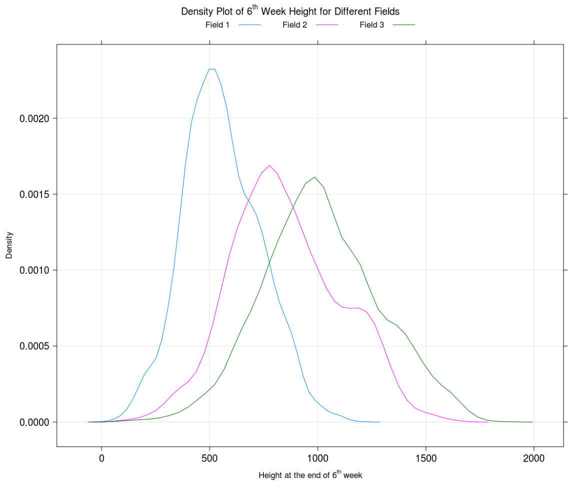
Density Plot of 6th Week Height for Different Fields
Field 1
Field 2
Field 3
Density
Height at the end of 6th week

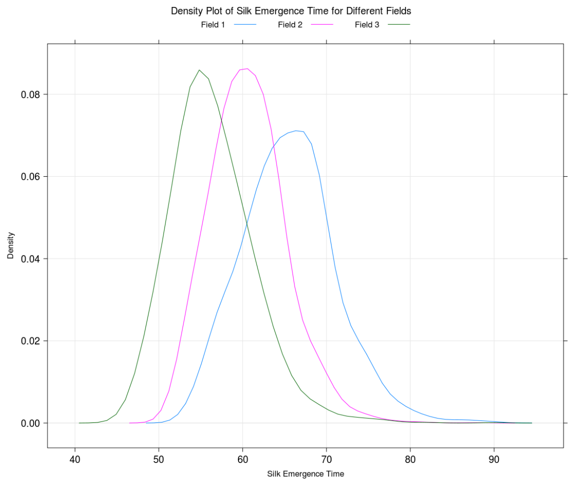
Density Plot of Silk Emergence Time for Different Fields
Field 1
Field 2
Field 3
Density
Silk Emergence Time

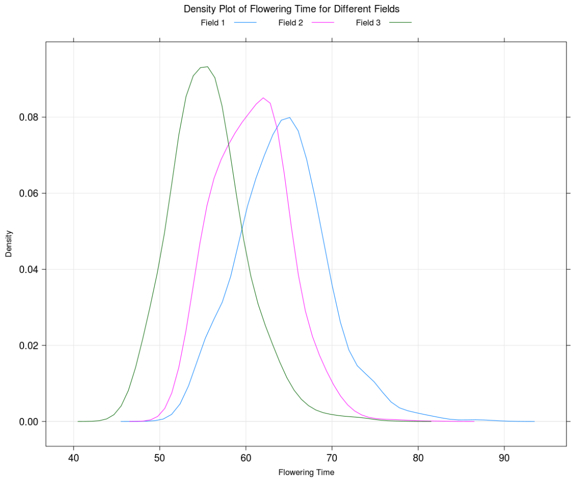
Density Plot of Flowering Time for Different Fields
Field 1
Field 2
Field 3
Density
Flowering Time

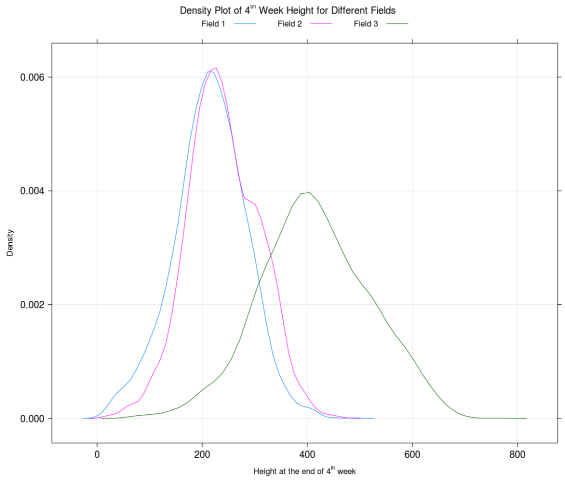
Density Plot of 4th Week Height for Different Fields
Field 1
Field 2
Field 3
Density
Height at the end of 4th week

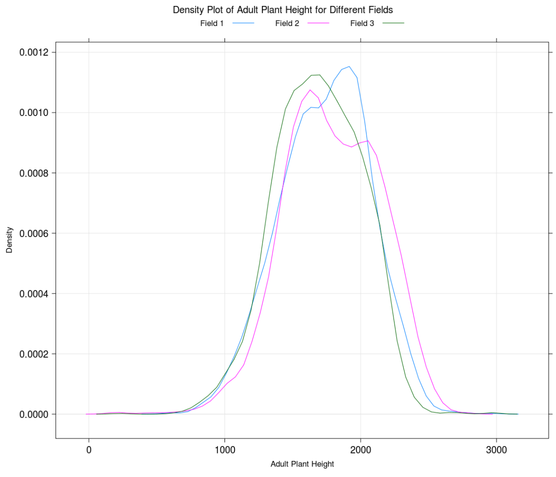
Density Plot of Adult Plant Height for Different Fields
Field 1
Field 2
Field 3
Density
Adult Plant Height

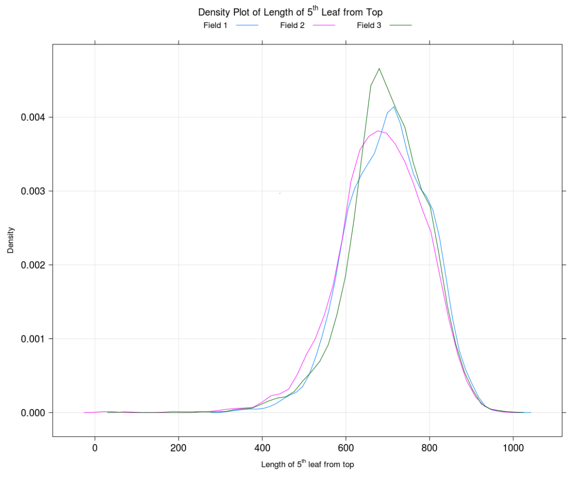
Density Plot of Length of 5th Leaf from Top
Field 1
Field 2
Field 3
Density
Length of 5th leaf from top

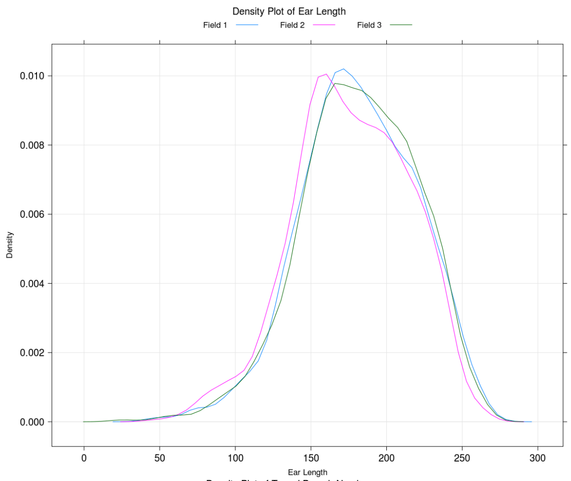

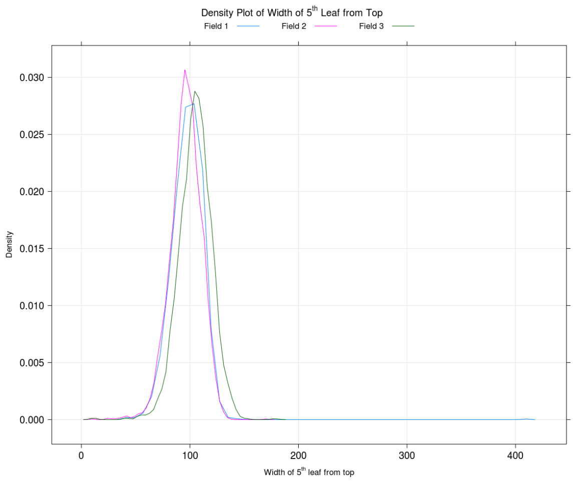

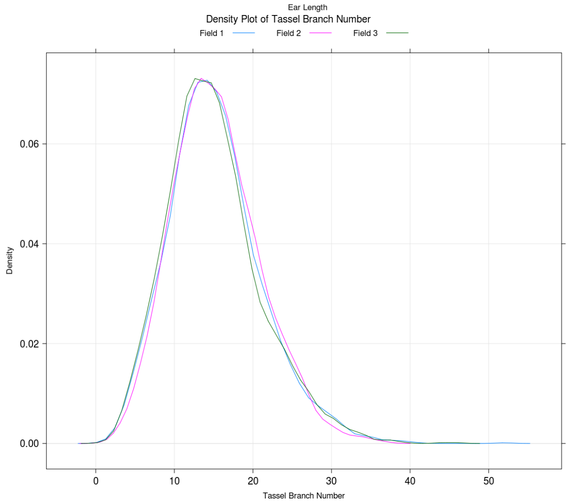

Figure 2.1: Density plots for all the phenotypes colored according to the field. The density plots are for 6th week height, silk emergence time, flowering time, 4th week height, adult plant height, length of 5th leaf from the top, ear length, width of the 5th leaf from the top and tassel branch number (left to right and top to bottom). They are arranged in a way that the heterogeneity in the data approximately decreases from the first to the last plot with respect to the fields.

We start the data analysis by visualizing the inbreeding depression rates for all the phenotypes based on the five different generations F1, S1, S3, S5 and S7. We further divided the plots based on the fields 1, 2 and 3 if the density plots in **Figure 2.1** showed heterogeneity for the corresponding phenotypic data. Inbreeding depression rates for 6th week height, silk emergence time, flowering time, and 4th week height were plotted separately for the three fields based on **Figure 2.1** as the phenotypic data from the three fields is very different.

We also found that there was an outlier in the phenotypic data for the width of the 5th leaf from the top; it was replaced by the median of the phenotypic data to make the plots clearer and the statistical analysis (in the **Methodology** section) free from its influence.

Figure 2.2 – Figure 2.5 show the distribution of inbreeding depression rates for 6th week height, silk emergence time, flowering time and 4th week height based on generation and field. **Figure 2.6 – Figure 2.10** show the distribution of inbreeding depression rates for the ear length, width of 5th leaf from top, length of 5th leaf from the top, adult height and the number of tassel branches.

The patterns in the inbreeding depression rates of the diploid plants and tetraploid plants are observed by looking at the red and blue box plots. The generation changes along the column from the F1 generation to S7 generation, from left to right and the fields change from 1 to 3, row-wise from top to bottom. The plots for F1 generation are not interesting as the data from this generation was used to calculate the depression rates for the siblings. It is included to check the quality of data, as dividing the phenotypic data of F1 generation by its observed mean should lead to a depression rate for F1 very close to 1. The inter-quartile ranges of most of the box plots for the F1 generations include 1, indicating an overall good quality of the data. Below are the details of the plots based on the depression rate of a particular phenotype.

The height of the plant at 6th week: (see: **Figure 2.2**)

The depression rate for the tetraploid plants **increases** in value from genotype A188/Oh43 to W22/B73. The pattern for the change in the depression rates for the genotypes is fairly consistent across generations for both ploidies. The box plots also show that the depression rate for the tetraploid W22/B73 genotype is different from the corresponding diploid plant. The depression rate of the diploid plants increases from A188/Oh43 to W22/B73, but the change is small compared to that of the tetraploid plants.

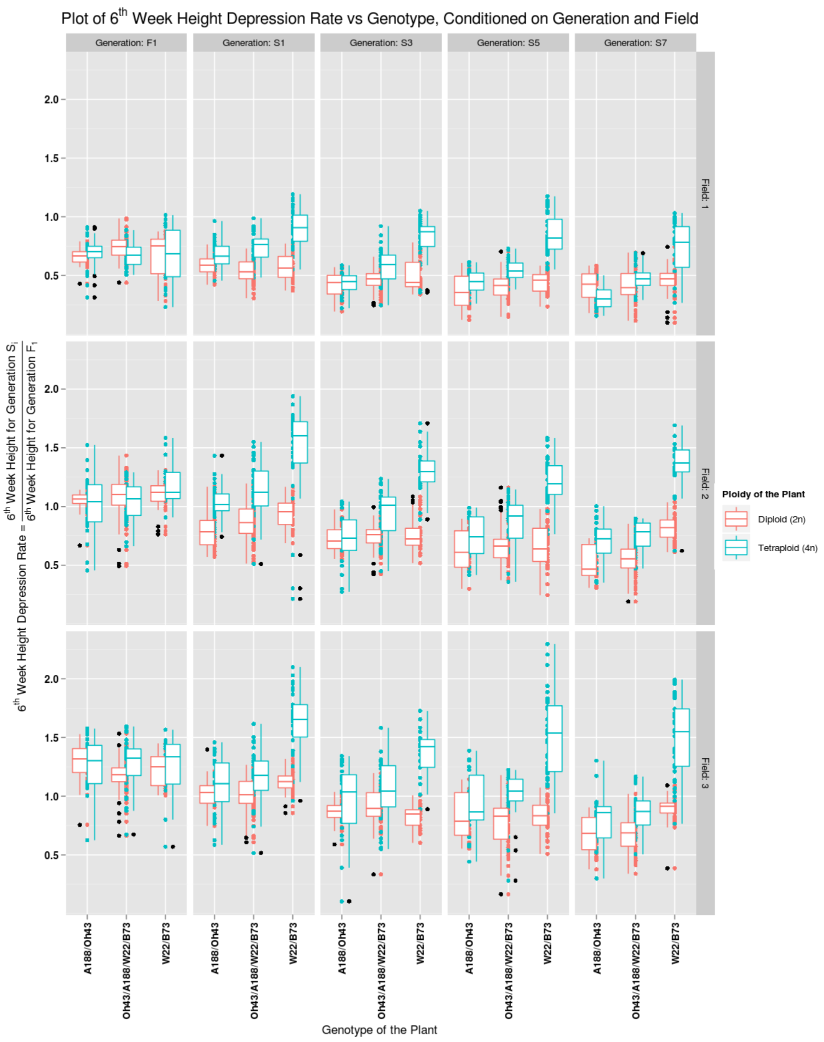

Figure 2.2: Plot of depression rate for 6th week height vs the genotype of the plant, conditioned on generations F1, S1, S3, S5 and S7 and fields 1, 2 and 3. The data for diploid plants are graphed in red and the tetraploid plants are graphed in blue. The patterns for a particular ploidy can be observed by looking at the box plots of the corresponding color. The generation varies from F1 to S7 column-wise and the fields vary from 1 to 3 row-wise.

The number of days to silk emergence after planting: (see: **Figure 2.3**)

The depression rates for both ploidies **decrease** from genotype A188/Oh43 to W22/B73. The pattern for the decrease in the depression rates is strongest for the S5 and S7 generation. The box plots also show that the depression rate for the W22/B73 tetraploid plants is different from corresponding diploid plants, across all generations.

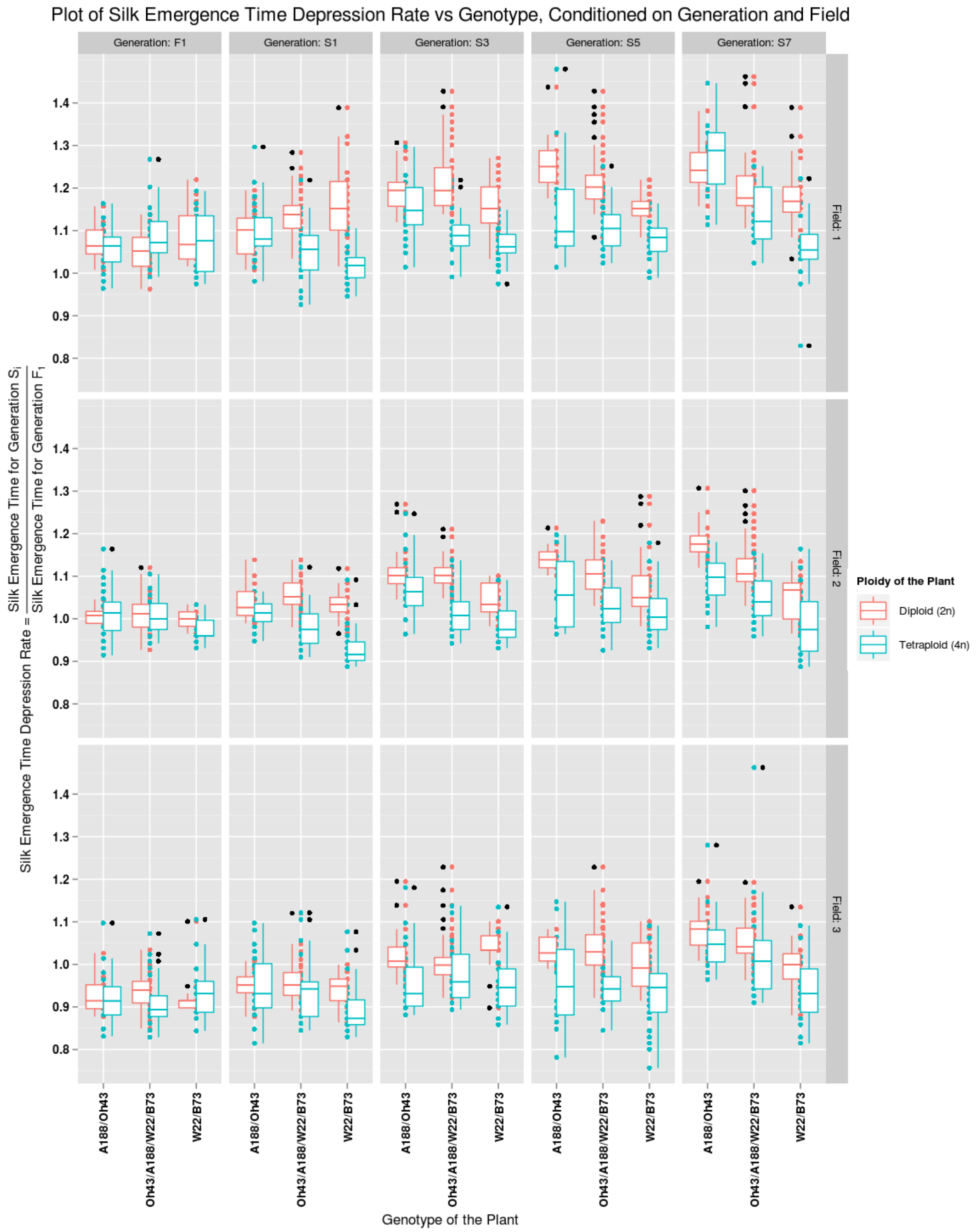

Figure 2.3: Plot of depression rate for silk time vs the genotype of the plant, conditioned on generations F1, S1, S3, S5 and S7 and fields 1, 2 and 3. The data for diploid plants are graphed in red and the tetraploid plants are graphed in blue. The patterns for a particular ploidy can be observed by looking at the box plots of the corresponding color. The generation varies from F1 to S7 column-wise and the fields vary from 1 to 3 row-wise.

The number of days to anther emergence after planting: (see: **Figure 2.4**)

The patterns in depression rates are similar to those observed in *silk emergence time*. In addition, the depression rates for W22/B73 are very different for the two ploidies across all generations.

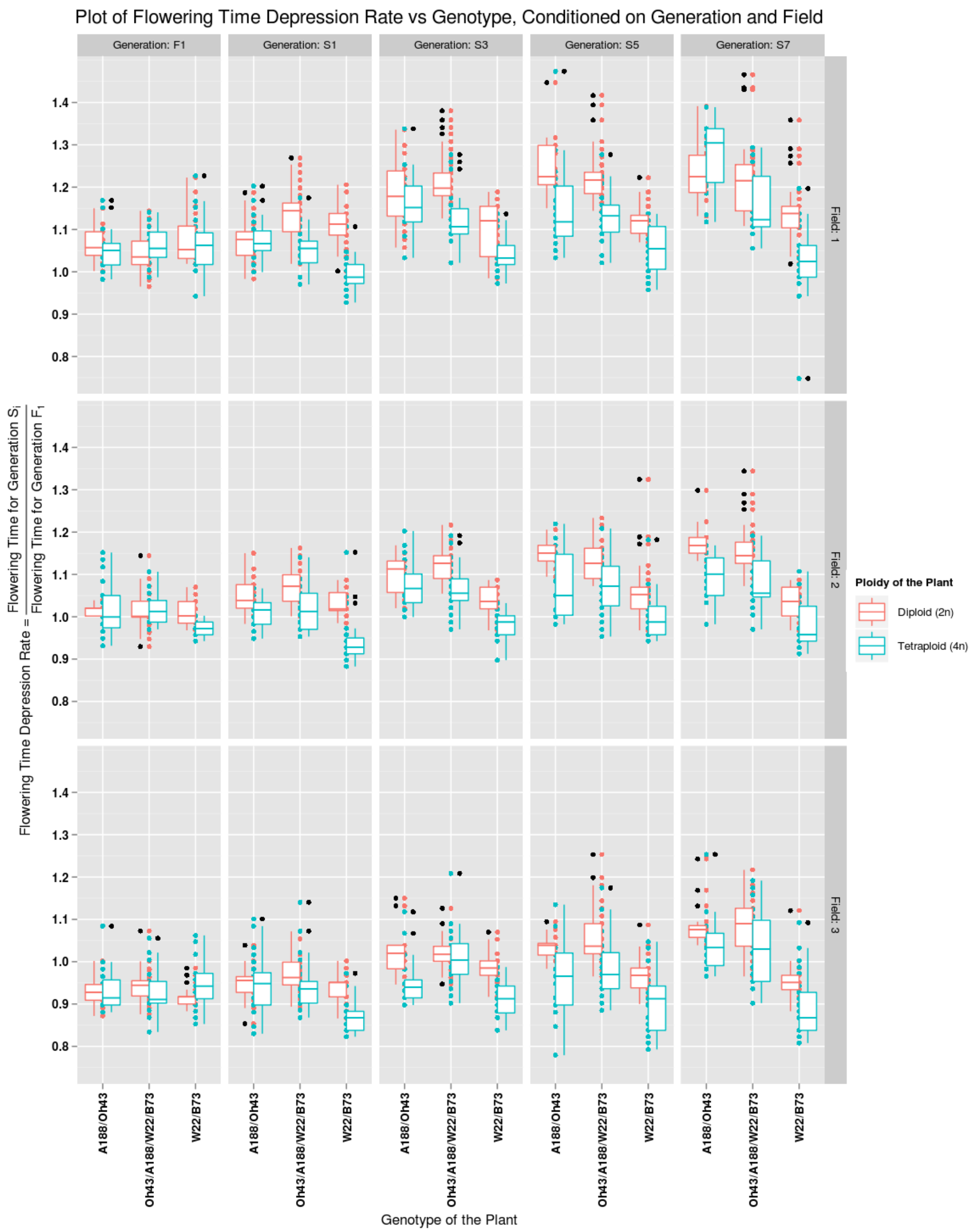

Figure 2.4: Plot of depression rate for flowering time vs the genotype of the plant, conditioned on generations F1, S1, S3, S5 and S7 and fields 1, 2 and 3. The data for diploid plants are graphed in red and the tetraploid plants are graphed in blue. The patterns for a particular ploidy can be observed by looking at the box plots of the corresponding color. The generation varies from F1 to S7 column-wise and the fields vary from 1 to 3 row-wise.

The height of the plant at 4th week: (see: **Figure 2.5**)

The patterns in depression rates are similar to those observed in *6th week plant height.*

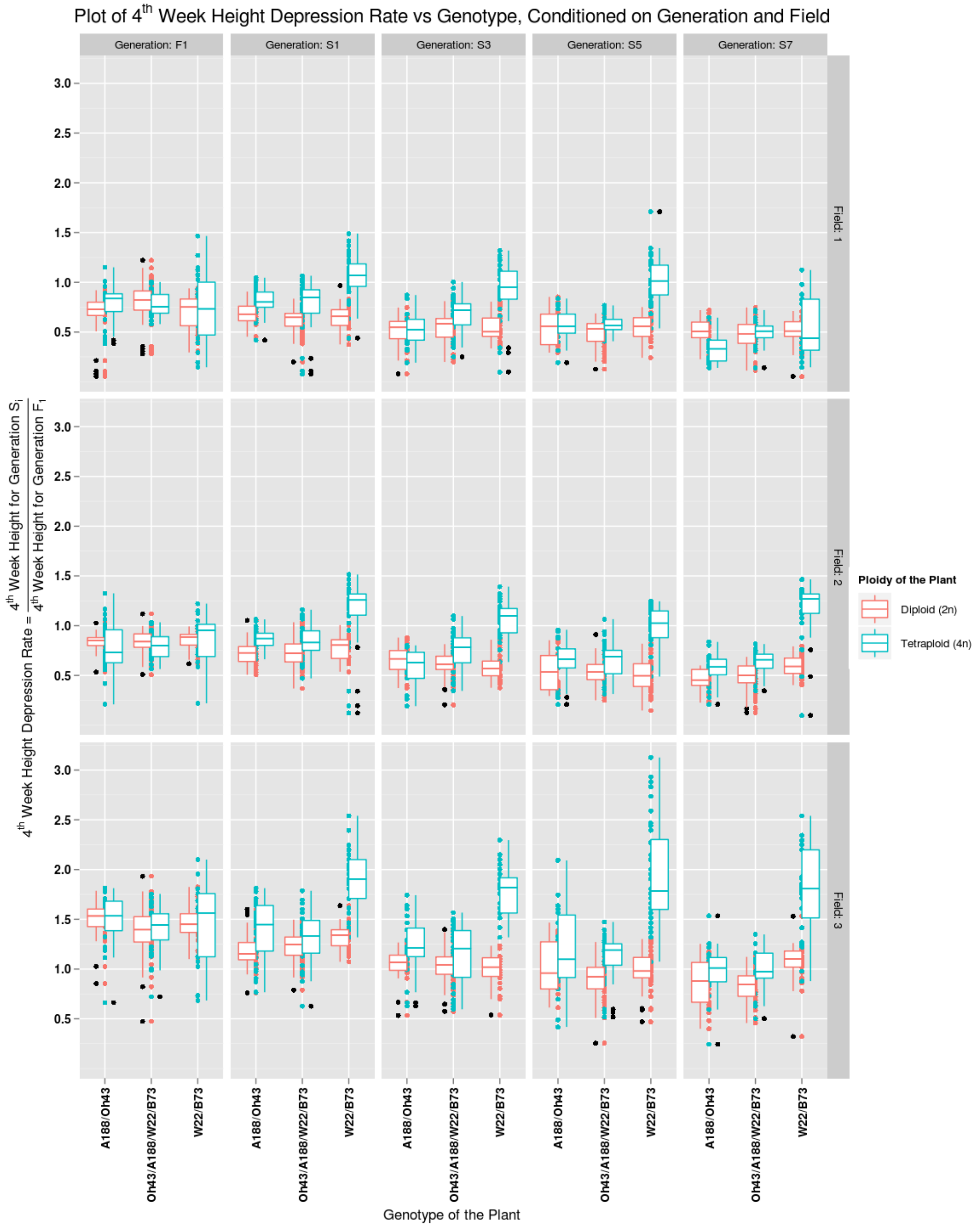

Figure 2.5: Plot of depression rate for 4th week height vs the genotype of the plant, conditioned on generations F1, S1, S3, S5 and S7 and fields 1, 2 and 3. The data for diploid plants are graphed in red and the tetraploid plants are graphed in blue. The patterns for a particular ploidy can be observed by looking at the box plots of the corresponding color. The generation varies from F1 to S7 column-wise and the fields vary from 1 to 3 row-wise.

The ear length of the maize plant: (see: **Figure 2.6**)

The depression rate **decreases** for the diploid plants and the pattern becomes stronger after generation S3. The tetraploid plants don't show any specific pattern, except the depression rate

is **lower** for A188/Oh43 x B73/W22 genotype in generations S5 and S7. There is no clear difference between the depression rates of diploid and tetraploid plants across all generations.

The width of the 5th leaf from the top: (see: **Figure 2.7**)

There are **no visible differences** between the depression rates of the diploid and the tetraploid plants. But we observe that the depression rates for the diploid plants are lower compared to the corresponding tetraploid plant.

The length of the 5th leaf from the top: (see: **Figure 2.8**)

The depression rate of the tetraploid plant **increases** across all generations from genotype A188/Oh43 to W22/B73. The depression rate for tetraploid B73/W22 genotype is higher than that of the diploid genotype; the pattern is strong after the S3 generation.

The height of the adult plant: (see: **Figure 2.9**)

The pattern in depression rates is similar to the pattern observed for the 6th week height. In addition, the diploid A188/Oh43 x B73/W22 genotype plants have **higher** depression rates compared to the other two diploid genotypes across all generations.

The tassel branch number: (see: **Figure 2.10**)

The diploid A188/Oh43 x B73/W22 genotype plants have a consistently **higher** depression rate where as the tetraploid A188/Oh43 x B73/W22 genotype plants have a consistently **lower** depression rate compared to the other two genotypes. It is also clear from the box plots that the depression rate for the diploid plant is higher than the tetraploid plant of this genotype.

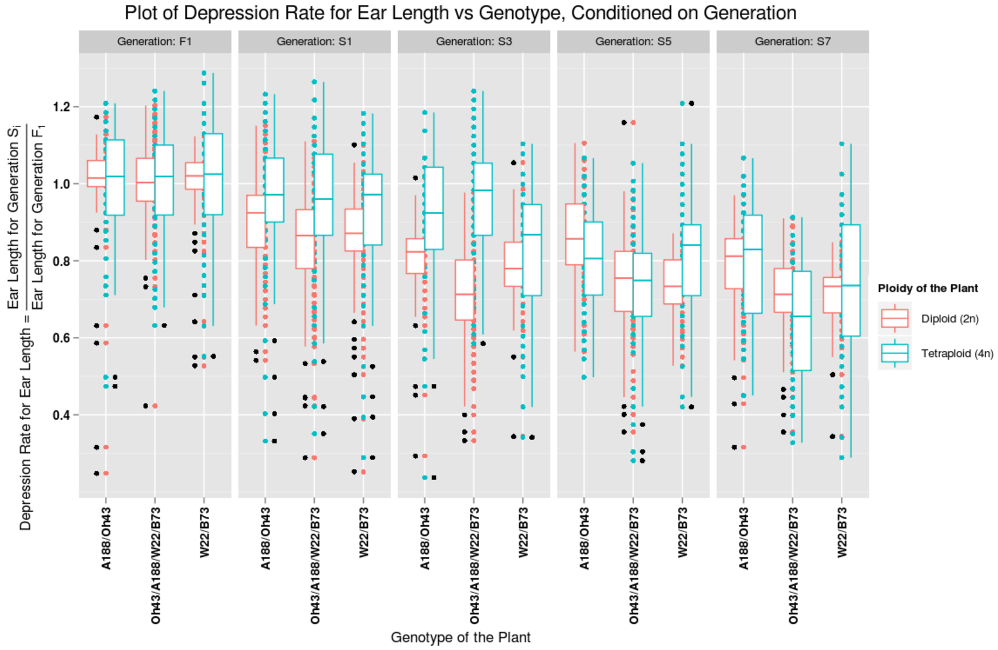

Figure 2.6: Plot of depression rate for ear length vs the genotype of the plant, conditioned on generations F1, S1, S3, S5 and S7. The conditioning on field is removed, as the data from all the three fields was shown to be homogenous by the density plots. The data for diploid plants are graphed in red and the tetraploid plants are graphed in blue. The patterns for a particular ploidy can be observed by looking at the box plots of the corresponding color. The generation varies from F1 to S7 column-wise.

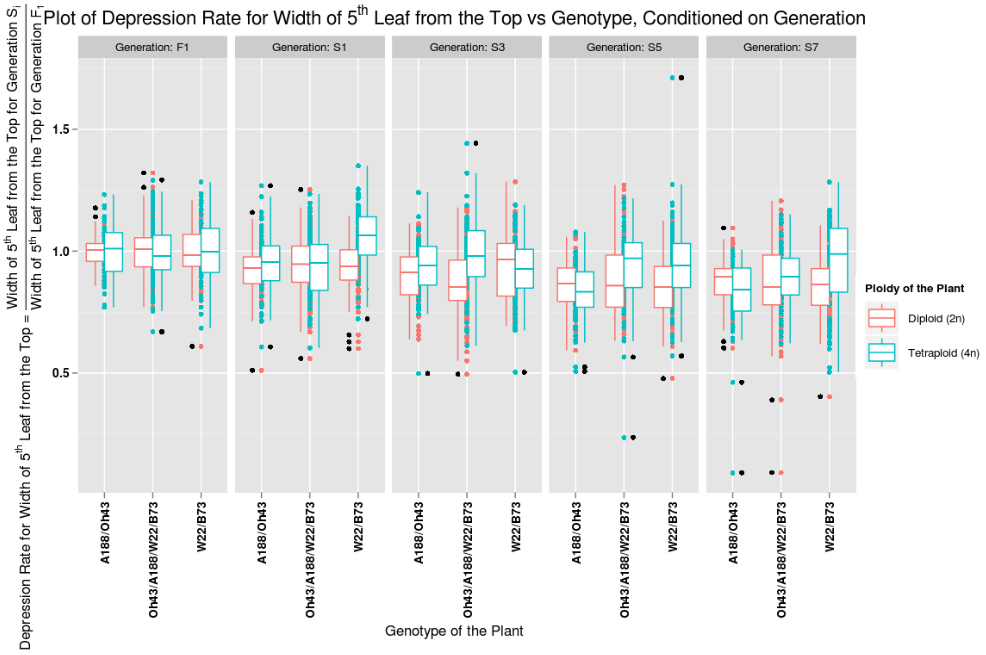

Figure 2.7: Plot of depression rate for width of 5th leaf vs the genotype of the plant, conditioned on generations F1, S1, S3, S5 and S7. The conditioning on field is removed, as the data from all the three fields was shown to be homogenous by the density plots. The data for diploid plants are graphed in red and the tetraploid plants are graphed in blue. The patterns for a particular ploidy can be observed by looking at the box plots of the corresponding color. The generation varies from F1 to S7 column-wise.

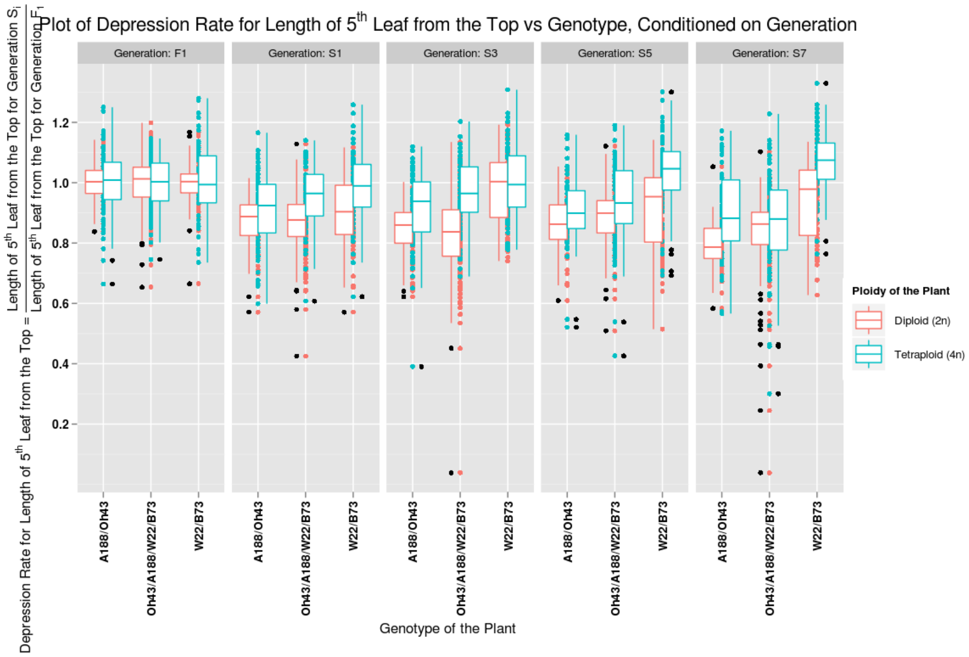

Figure 2.8: Plot of depression rate for length of 5th leaf vs the genotype of the plant, conditioned on generations F1, S1, S3, S5 and S7. The conditioning on field is removed, as the data from all the three fields was shown to be homogenous by the density plots. The data for diploid plants are graphed in red and the tetraploid plants are graphed in blue. The patterns for a particular ploidy can be observed by looking at the box plots of the corresponding color. The generation varies from F1 to S7 column-wise.

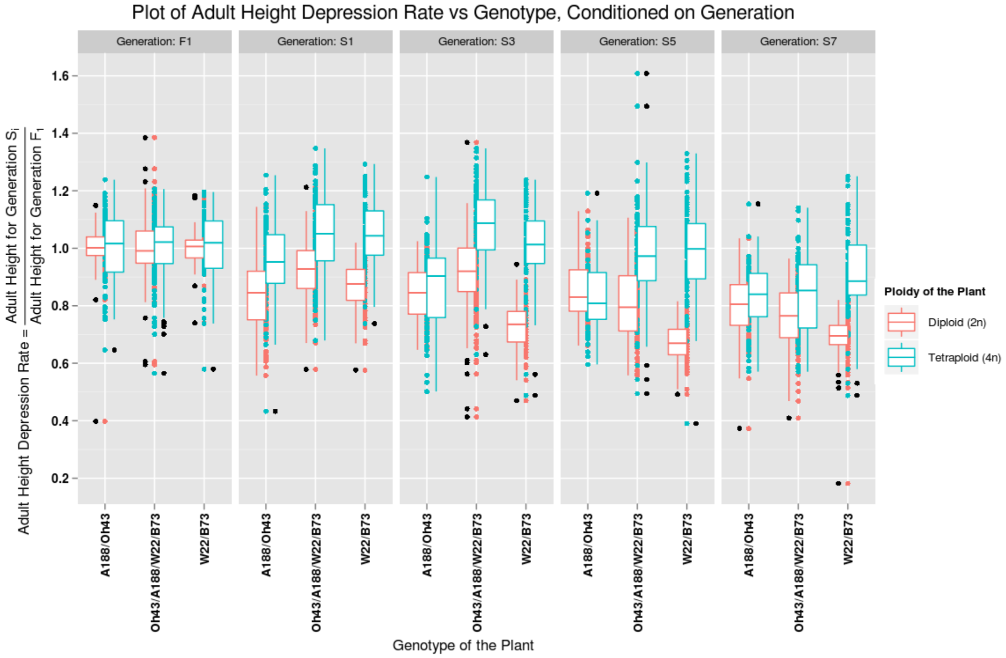

Figure 2.9: Plot of depression rate for adult height vs the genotype of the plant, conditioned on generations F1, S1, S3, S5 and S7. The conditioning on field is removed, as the data from all the three fields was shown to be homogenous by the density plots. The data for diploid plants are graphed in red and the tetraploid plants are graphed in blue. The patterns for a particular ploidy can be observed by looking at the box plots of the corresponding color. The generation varies from F1 to S7 column-wise.

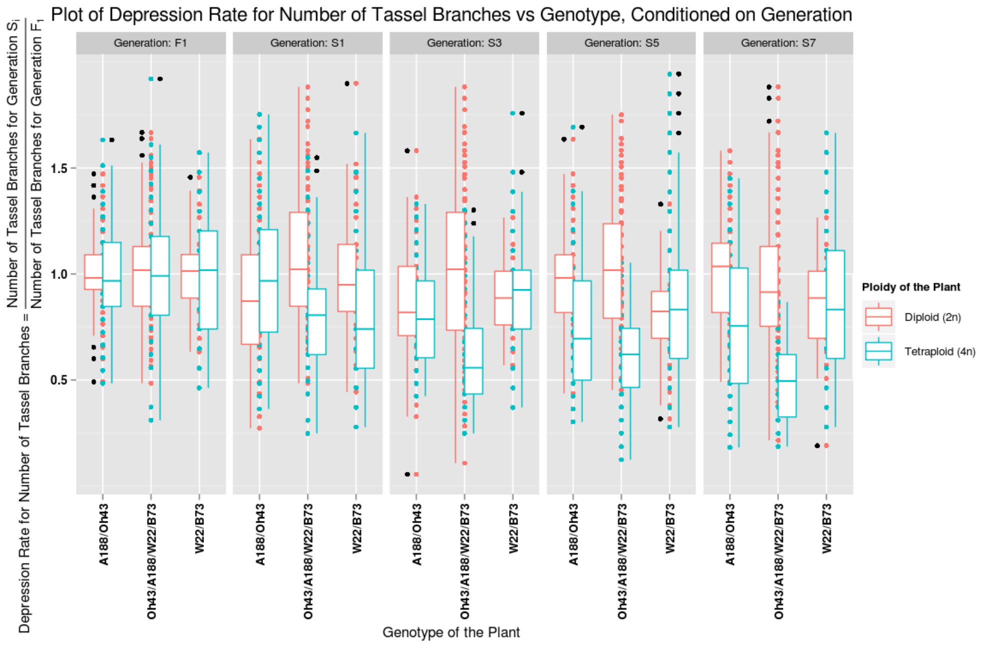

Figure 2.10: Plot of depression rate for the number of tassel branches vs the genotype of the plant, conditioned on generations F1, S1, S3, S5 and S7. The conditioning on field is removed, as the data from all the three fields was shown to be homogenous by the density plots. The data for diploid plants are graphed in red and the tetraploid plants are graphed in blue. The patterns for a particular ploidy can be observed by looking at the box plots of the corresponding color. The generation varies from F1 to S7 column-wise.

3. METHODOLOGY – ANALYSIS OF VARIANCE (ANOVA)

The details of the analysis that provided answers to questions *1 –7* in **Section 1** are:

1. **Is the inbreeding depression rate different between diploid and tetraploid lines with the same genetic constitution?**

An analysis of variance (ANOVA) method was employed to test for differences between inbreeding depression rates of diploid and tetraploid lines with the same genetic constitution. The statistical model is:

$$Depression\ Rate_{ijklm} = Generation_i + Genotype_j + Ploidy_k + (Generation \times Genotype)_{ij} + (Generation \times Ploidy)_{ik} + (Genotype \times Ploidy)_{jk} + (Generation \times Genotype \times Ploidy)_{ijk} + Field_l + \varepsilon_{ijklm}$$

i = 1, 2, 3, 4, 5 (corresponds to F1, S1, S3, S5, S7 generations)
j = 1, 2, 3 (corresponds to A188/Oh43, Oh43/A188/W22/B73, W22/B73 genotypes)
k = 1, 2 (corresponds to diploid and tetraploid plants)
l = 1, 2, 3 (corresponds to the blocks)
m= 1 … 12 (at most) (corresponds to the biological replicates) (2)

$Field_l$ is a random effect and is assumed to be distributed normally with mean 0 and variance σ^2_{field}. ε_{ijklm} is the error term and is assumed to be distributed normally with mean 0 and variance σ^2_{error}. Every other term is considered fixed effect and the names of the variable correspond to the variables in the statistical analysis. The terms with two or more variable names are the corresponding interaction terms.

The ANOVA model (2) is for a randomized complete block design with field as the block and generation, genotype and ploidy as the treatment factors. The question of interest can be answered by testing the following hypotheses:

$\mathbf{H_o}$: $(Generation \times Genotype \times Ploidy)_{ijk} = 0$ for a given value of i and j for k = 1 and 2. (Inbreeding depression rate is not different between diploid and tetraploid lines with the same genetic constitution)

$\mathbf{H_a}$: $(Generation \times Genotype \times Ploidy)_{ijk} \neq 0$ for a given value of i and j for k = 1 and 2. (Inbreeding depression rate is different between diploid and tetraploid lines with the same genetic constitution) (H1)

i = 1, 2, 3, 4, 5 (corresponds to F1, S1, S3, S5, S7 generations)
j = 1, 2, 3 (corresponds to A188/Oh43, Oh43/A188/W22/B73, W22/B73 genotypes)
k = 1, 2 (corresponds to diploid and tetraploid plants)

To test the hypotheses for the interaction of generation, genotype and ploidy at a particular genotype and generation, we will use the *slice* option in the *lsmeans* command of the SAS MIXED PROCEDURE. These hypotheses are tested using likelihood ratio test (LRT) statistic estimated by the Maximum Likelihood procedure. The LRT statistic is distributed as χ^2 with 1 degree of freedom under the null hypothesis (Section 8.2, Faraway, 2006). We used a significance level of 0.05 and did not correct for multiple comparison as most of the p-values were lower than 0.0001. The details of the SAS code are provided in the appendix section. The results are summarized in the **Results** section.

2. **Is the inbreeding depression rate different between lines with different genetic constitution but the same ploidy?**

An analysis of variance (ANOVA) method was employed to detect the difference between inbreeding depression rate with different genetic constitution for diploid and tetraploid plants separately. The statistical model for this problem is:

$$Depression\ Rate_{ijlm} = Generation_i + Genotype_j + (Generation \times Genotype)_{ij} + Field_l + \varepsilon_{ijlm}$$

i = 1, 2, 3, 4, 5 (corresponds to F1, S1, S3, S5, S7 generations) for diploid and tetraploid plants.

j = 1, 2, 3 (corresponds to A188/Oh43, Oh43/A188/W22/B73, W22/B73 genotypes) for the tetraploid plants.

j = 1 … 14 (corresponds to Oh43/A188, A188/Oh43, W22/B73, B73/W22, Oh43/W22, W22/Oh43, W22/A188, A188/W22, B73/A188, A188/B73, B73/Oh43, Oh43/B73, A188/Oh43 x B73/W22, B73/W22 x A188/Oh43 genotypes) for the diploid plants.

l = 1, 2, 3 (corresponds to the blocks) for diploid and tetraploid plants.

m= 1 … 12 (at most) (corresponds to the biological replicates) for diploid and tetraploid plants

(3)

$Field_l$ is a random effect and is assumed to be distributed normally with mean 0 and variance σ^2_{field}. ε_{ijklm} is the error term and is assumed to be distributed normally with mean 0 and variance σ^2_{error}. Every other term is considered fixed effect and the names of the variable correspond to the variables in the statistical analysis. The terms with two variable names are the corresponding interaction terms.

The above ANOVA model is for a randomized complete block design with field as the block and generation and genotype as the treatment factors. The question of interest can be answered by testing the following hypotheses:

For the diploid plants:

H_o: $(Generation \times Genotype)_{ij} = 0$ for a given value of i and j= 1 … 14 (Inbreeding depression rate is not different between lines with different genetic constitution within diploid plants.)

H_a: $(Generation \times Genotype)_{ij} \neq 0$ for a given value of i and j= 1 … 14 (Inbreeding depression rate is different between lines with different genetic constitution within tetraploid plants.)

For the tetraploid plants:

H_o: $(Generation \times Genotype)_{ij} = 0$ for a given value of i and j= 1, 2, 3 (Inbreeding depression rate is not different between lines with different genetic constitution within diploid plants.)

H_a: $(Generation \times Genotype)_{ij} \neq 0$ for a given value of i and j= 1, 2, 3 (Inbreeding depression

rate is different between lines with different genetic constitution within tetraploid plants.)

(H2)

The conventions followed for i and j are same as in the model (3).

To test the hypotheses for the interaction of generation and genotype for a fixed ploidy of different generations, we will use the *slice* option in the *lsmeans* command of the SAS MIXED PROCEDURE. These hypotheses are tested using likelihood ratio test (LRT) statistic estimated by the Maximum Likelihood procedure. The LRT statistic is distributed as χ^2 with 1 degree of freedom under the null hypothesis (Section 8.2, Faraway, 2006). We used a significance level of 0.05 and did not correct for multiple comparison as most of the p-values were lower than 0.0001. The details of the SAS code are provided in the appendix section. The results are summarized in the **Results** section.

3. Does inbreeding depression occur in all the measured phenotypes?

The SAS output from the hypothesis testing procedure in the first and second questions give the answer to this question. The question of interest can be answered by testing the following hypotheses for all the phenotypes:

.

H₀: $Generation_i = Genotype_j = Ploidy_k = (Generation \times Genotype)_{ij} = (Generation \times Ploidy)_{ik} = (Genotype \times Ploidy)_{jk} = (Generation \times Genotype \times Ploidy)_{ijk} = 0$

Hₐ: $Generation_i = Genotype_j = Ploidy_k = (Generation \times Genotype)_{ij} = (Generation \times Ploidy)_{ik} = (Genotype \times Ploidy)_{jk} = (Generation \times Genotype \times Ploidy)_{ijk} \neq 0$

(H3)

i = 1, 2, 3, 4, 5 (corresponds to F1, S1, S3, S5, S7 generations)
j = same as in the model (3) (corresponds to all genotypes)
k = 1, 2 (corresponds to diploid and tetraploid plants)

4. Is there depression in every diploid and tetraploid genotype?

Similar to the third question, the analysis and SAS output of the second question answers this question. The question of interest can be answered by testing the following hypotheses (same as questions 2) for all the phenotypes:

For the diploid plants:

H_o: $Generation_i = Genotype_j = Ploidy_k = (Generation \times Genotype)_{ij} = (Generation \times Ploidy)_{ik} = (Genotype \times Ploidy)_{jk} = (Generation \times Genotype \times Ploidy)_{ijk} = 0$

H_a: $Generation_i = Genotype_j = Ploidy_k = (Generation \times Genotype)_{ij} = (Generation \times Ploidy)_{ik} = (Genotype \times Ploidy)_{jk} = (Generation \times Genotype \times Ploidy)_{ijk} \neq 0$

For the tetraploid plants:

H_o: $Generation_i = Genotype_j = Ploidy_k = (Generation \times Genotype)_{ij} = (Generation \times Ploidy)_{ik} = (Genotype \times Ploidy)_{jk} = (Generation \times Genotype \times Ploidy)_{ijk} = 0$

H_a: $Generation_i = Genotype_j = Ploidy_k = (Generation \times Genotype)_{ij} = (Generation \times Ploidy)_{ik} = (Genotype \times Ploidy)_{jk} = (Generation \times Genotype \times Ploidy)_{ijk} \neq 0$

(H4)

The conventions followed for i and j are same as in the model (3).

5. **How is the inbreeding depression rate affected by ploidy, genetic constitution and the interaction between ploidy and genetic constitution?**

The SAS output for the first question gives the p-values for individual factors and for all the possible interactions between the factors. The question of interest can be answered by testing the following hypotheses for all the phenotypes:

H_o: $(Generation \times Genotype \times Ploidy)_{ijk} = 0$

H_a: $(Generation \times Genotype \times Ploidy)_{ijk} \neq 0$

(H5)

i = 1, 2, 3, 4, 5 (corresponds to F1, S1, S3, S5, S7 generations)

j = 1, 2, 3 (corresponds to A188/Oh43, Oh43/A188/W22/B73, W22/B73 genotypes)

$k = 1, 2$ (corresponds to diploid and tetraploid plants)

6. **Are there any parental effects on inbreeding depression rate?**

An analysis of variance (ANOVA) method was employed to test for differences between inbreeding depression rate of reciprocal crosses in the diploid plants.

$$Depression\ Rate_{ijlm} = Generation_i + Genotype_j + (Generation \times Genotype)_{ij} + Field_l + \varepsilon_{ijlm}$$

$i = 1, 2, 3, 4, 5$ (corresponding to F1, S1, S3, S5, S7 generations)
j = 1, 2 (corresponding to the reciprocal crosses, for example: Oh43/A188 and A188/Oh43)
$l = 1, 2, 3$ (corresponding to the blocks)
m= 1 … 12 (at most) (corresponding to the biological replicates) for diploid plants

(4)

$Field_l$ is a random effect and is assumed to be distributed normally with mean 0 and variance σ^2_{field}. ε_{ijklm} is the error term and is assumed to be distributed normally with mean 0 and variance σ^2_{error}. Every other term is considered fixed effect and the names of the variable correspond to the variables in the statistical analysis. The terms with two variable names are the corresponding interaction terms.

This ANOVA model is fit all seven reciprocal crosses (listed below) separately:
Oh43/A188 and A188/Oh43
W22/B73 and B73/W22
Oh43/W22 and W22/Oh43
W22/A188 and A188/W22
B73/A188 and A188/B73
B73/Oh43 and Oh43/B73
A188/Oh43 x B73/W22
B73/W22 x A188/Oh43.

The ANOVA model (4) is for a randomized complete block design with field as the block and

generation and genotype as the treatment factors. In order to answer the question of interest we will test the following hypotheses:

For a particular reciprocal cross:

H₀: $(Generation \times Genotype)_{ij} = 0$ for a given value of i and j= 1, 2 (Inbreeding depression rate is not different between reciprocal crosses.)

Hₐ: $(Generation \times Genotype)_{ij} \neq 0$ for a given value of i and j= 1, 2 (Inbreeding depression rate is different between reciprocal crosses.) (H6)

The conventions followed for i and j are same as in the model (4).

To test the hypotheses for the interaction of generation and genotype for a particular reciprocal cross at different generations, we will use the *slice* option in the *lsmeans* command of the SAS MIXED PROCEDURE. These hypotheses are tested using likelihood ratio test (LRT) statistic estimated by the Maximum Likelihood procedure. The LRT statistic is distributed as χ^2 with 1 degree of freedom under the null hypothesis (Section 8.2, Faraway, 2006). We used a significance level of 0.05 and did not correct for multiple comparison as most of the p-values were lower than 0.0001. The details of the SAS code are provided in the appendix section. The results are summarized in the **Results** section.

7. Are the S7 lines different from their corresponding progenitor inbred lines?

An analysis of variance (ANOVA) method was employed to detect the difference between the phenotypic value of the S7 line and the corresponding inbred lines.

$$phenotype_{jlm} = Genotype_j + Field_l + \varepsilon_{jlm}$$

j = 1, 2 (corresponds to S7 and one of the two parent)

l = 1, 2, 3 (corresponds to the blocks)

m= 1 … 12 (at most) (corresponds to the biological replicates) (5)

$Field_l$ is a random effect and is assumed to be distributed normally with mean 0 and variance σ^2_{field}. ε_{ijklm} is the error term and is assumed to be distributed normally with mean 0 and variance σ^2_{error}. Genotype$_i$ (S7 or parent) is considered a fixed effect.

The ANOVA model (5) is fit to compare any S7 genotype with its parents separately. This is done for all the genotypes in both the ploidies. In order to answer the question of interest we will test the following hypotheses:

For a particular genotype, parent, phenotype and ploidy:
H$_o$: $Genotype_j = 0$ (S7 line is not different from the corresponding inbred lines)
H$_a$: $Genotype_j \neq 0$ (S7 line is different from the corresponding inbred lines) (H7)

The conventions followed for j is same as in the model (5).

We used the SAS MIXED PROCEDURE to fit model with Genotype as the fixed effect and field as random for all the phenotype and ploidies. These hypotheses are tested using likelihood ratio test (LRT) statistic estimated by the Maximum Likelihood procedure. The LRT statistic is distributed as χ^2 with 1 degree of freedom under the null hypothesis (Section 8.2, Faraway, 2006). We used a significance level of 0.05 and did not correct for multiple comparison as most of the p-values were lower than 0.0001. The details of the SAS code are provided in the appendix section. There were a total of forty comparisons. The results are summarized in the **Results** section.

4. Results

The summary of the results for all the questions are as follows. In all the summaries below *Yes* denotes that the null hypothesis was rejected in favor of the alternative and ***No*** denotes that there was not enough evidence to reject the null hypothesis (i.e., significance level is 5%).

1. **Is the inbreeding depression rate different between diploid and tetraploid lines with the same genetic constitution?**

We assumed that the tetraploid Oh43/A188/W22/B73 genotype was identical to A188/Oh43 x B73/W22 and B73/W22 x A188/Oh43 diploid genotypes. In the table below, in a particular phenotypic column, *Yes* denotes that the null hypothesis was rejected in favor of the alternative and ***No*** denotes that there was not enough evidence to reject the null hypothesis for that phenotype (see: **Section 3, H1**).

**Generation**	**Genotype (Maize Lines)**	**Height at the end of 4 weeks**	**Height at the end of 6 week**	**Adult Height**	**Flowering time**	**Silk Time**	**Length of the 5th leaf**	**Width of the 5th leaf**	**No. of Tassel Braches**	**Ear Length**
F1	A188/Oh43	**No**	**No**	**No**	**No**	**No**	**No**	**No**	**No**	**No**
F1	Oh43/A188/W22/B73	**No**	**No**	**No**	**No**	**No**	**No**	**No**	**No**	**No**
F1	W22/ B73	**No**	**No**	**No**	**No**	**No**	**No**	**No**	**No**	**No**
S1	A188/Oh43	Yes	Yes	Yes	Yes	**No**	Yes	Yes	**No**	Yes
S1	Oh43/A188/W22/B73	Yes	Yes	Yes	Yes	Yes	Yes	**No**	Yes	Yes
S1	W22/ B73	Yes	Yes	Yes	Yes	Yes	Yes	Yes	Yes	Yes
S3	A188/Oh43	**No**	**No**	**No**	Yes	Yes	Yes	Yes	**No**	Yes
S3	Oh43/A188/W22/B73	Yes	Yes	Yes	Yes	Yes	Yes	Yes	Yes	Yes
S3	W22/ B73	Yes	Yes	Yes	Yes	Yes	**No**	**No**	**No**	**No**
S5	A188/Oh43	Yes	Yes	**No**	Yes	Yes	Yes	**No**	Yes	Yes
S5	Oh43/A188/W22/B73	Yes	Yes	Yes	Yes	Yes	Yes	Yes	Yes	**No**
S5	W22/ B73	Yes	Yes	Yes	Yes	Yes	Yes	Yes	**No**	Yes
S7	A188/Oh43	**No**	Yes	**No**	Yes	Yes	Yes	Yes	Yes	**No**
S7	Oh43/A188/W22/B73	Yes	Yes	Yes	Yes	Yes	**No**	**No**	Yes	Yes
S7	W22/ B73	Yes	Yes	Yes	Yes	Yes	Yes	Yes	**No**	**No**

2. **Is the inbreeding depression rate different between lines with different genetic constitution but the same ploidy?**

All the genotypic effects of tetraploid and diploid lines were significant except the genotypic effect of tetraploid lines in the S1 generation was not significant for the ear length phenotype.

3. **Does inbreeding depression occur in all the measured phenotypes?**

Inbreeding depression occurs in all the measured phenotypes. The results for a particular generation and genotype are summarized in the results of first question for all the phenotypes. The results for a particular generation and ploidy are summarized in the results of second question for all the phenotypes.

4. **Is there depression in every diploid and tetraploid genotype?**

There is inbreeding depression in all the genotypes and in both the ploidies across all siblings.

5. **How is the inbreeding depression rate affected by ploidy, genetic constitution and the interaction between ploidy and genetic constitution?**

Inbreeding depression rate is affected by ploidy, genetic constitution and the interaction between ploidy and genetic constitution.

6. **Are there any parental effects on inbreeding depression rate?**

Following table summarizes the results of statistical hypotheses tests (**Section 3, H6**) to determine the difference of inbreeding depression rate between reciprocal pairs based on generations F1, S1, S3, S5 and S7 and for all the phenotypes. For a particular phenotype, in the table below, row represents the generation and the columns denote one of the seven reciprocal crosses. *Yes* denotes that the null hypothesis was rejected in favor of the alternative and ***No*** denotes that there was not enough evidence to reject the null hypothesis (**Section 3, H6**).

Generation	AB	AO	AW	ABOW	BO	BW	OW
**Height at the end of 4th Week**							
**F1**	**No**	**No**	**No**	**No**	**No**	**No**	**No**
**S1**	**No**	**No**	**No**	Yes	Yes	**No**	Yes
**S3**	**No**	Yes	**No**	**No**	Yes	**No**	Yes
**S5**	**No**	Yes	**No**	Yes	**No**	**No**	Yes
**S7**	Yes	Yes	Yes	Yes	Yes	**No**	**No**
**Height at the end of 6th Week**							
**F1**	**No**	**No**	**No**	**No**	**No**	**No**	**No**
**S1**	Yes	**No**	**No**	Yes	**No**	**No**	**No**
**S3**	**No**	Yes	**No**	Yes	**No**	**No**	Yes
**S5**	**No**	Yes	**No**	Yes	**No**	Yes	**No**
**S7**	**No**	**No**	**No**	Yes	Yes	**No**	**No**
**Adult Plant Height**							
**F1**	**No**	**No**	**No**	**No**	**No**	**No**	**No**
**S1**	**No**	Yes	**No**	Yes	**No**	Yes	**No**
**S3**	**No**	Yes	Yes	Yes	Yes	Yes	Yes
**S5**	**No**	Yes	**No**	Yes	Yes	Yes	**No**
**S7**	**No**	Yes	**No**	Yes	Yes	Yes	Yes
**Number of Days to Emergence of Anthers**							
**F1**	**No**	**No**	**No**	**No**	**No**	**No**	**No**
**S1**	**No**	**No**	**No**	Yes	Yes	**No**	**No**
**S3**	Yes	**No**	**No**	Yes	**No**	Yes	Yes
**S5**	Yes	**No**	**No**	Yes	Yes	**No**	**No**
**S7**	Yes	Yes	**No**	Yes	**No**	Yes	Yes

**Number of Days to Emergence of Silk**							
**F1**	**No**	**No**	**No**	**No**	**No**	**No**	**No**
**S1**	Yes	**No**	**No**	Yes	Yes	**No**	**No**
**S3**	Yes	Yes	Yes	**No**	Yes	**No**	Yes
**S5**	Yes	Yes	**No**	**No**	Yes	**No**	**No**
**S7**	Yes	**No**	Yes	Yes	**No**	**No**	Yes
**Length of the 5th Leaf from the Top**							
**F1**	**No**	**No**	**No**	**No**	**No**	**No**	**No**
**S1**	**No**	**No**	**No**	Yes	**No**	**No**	**No**
**S3**	Yes	**No**	**No**	Yes	**No**	Yes	Yes
**S5**	Yes	Yes	Yes	**No**	Yes	Yes	**No**
**S7**	Yes	**No**	**No**	Yes	**No**	Yes	**No**
**Width of the 5th Leaf from the Top**							
**F1**	**No**	**No**	**No**	**No**	**No**	**No**	**No**
**S1**	Yes	Yes	Yes	Yes	Yes	Yes	**No**
**S3**	Yes	**No**	**No**	Yes	Yes	**No**	**No**
**S5**	Yes	**No**	**No**	Yes	Yes	Yes	Yes
**S7**	Yes	**No**	**No**	Yes	Yes	Yes	**No**
**Number of Tassel Branches**							
**F1**	**No**	**No**	**No**	**No**	**No**	**No**	**No**
**S1**	**No**	**No**	**No**	Yes	Yes	Yes	**No**
**S3**	Yes	Yes	Yes	Yes	Yes	Yes	Yes
**S5**	**No**	Yes	**No**	Yes	Yes	Yes	**No**
**S7**	Yes	Yes	**No**	Yes	Yes	**No**	Yes
**Ear Length**							
**F1**	**No**	**No**	**No**	**No**	**No**	**No**	**No**

**S1**	**No**	**No**	**No**	Yes	**No**	Yes	**No**
**S3**	**No**	Yes	**No**	**No**	Yes	Yes	Yes
**S5**	Yes	Yes	Yes	Yes	Yes	Yes	Yes
**S7**	**No**	Yes	**No**	**No**	Yes	Yes	Yes

7. **Are the S7 lines different from their corresponding inbred lines?**

Following table summarizes the results of statistical hypotheses tests (**Section 3, H7**) to determine the difference of phenotypes between the S7 generation and corresponding inbred lines for the diploid and tetraploid plants. In the table below, row represents the phenotype and the columns contain the hypotheses test result corresponding to S7 and one of its parents for all the genotypes. There are separate tables for tetraploid and diploid plants as the genotypes differ between the two ploidies. The column labels have the naming scheme: S7 genotype -- Parent genotype.

For the tetraploid plants:

Genotype (column)/ Phenotype (row)	A188/ O43--A188	A188/ O43 - - O43	W22/ B73--B73	W22/ B73--W22	O43/ A188/ W22/B73 --A188	O43/ A188/ W22/B73 -- O43	O43/ A188/ W22/B73 -- B73	O43/ A188/ W22/B73 -- W22
4th Week Height	Yes	**No**	Yes	Yes	Yes	Yes	Yes	Yes
6th Week Height	Yes	**No**	Yes	Yes	Yes	Yes	Yes	Yes
Adult Height	Yes	Yes	Yes	Yes	Yes	Yes	Yes	Yes
Flower Time	Yes	**No**	Yes	**No**	**No**	Yes	Yes	Yes
Silk Time	**No**	**No**	Yes	**No**	**No**	Yes	Yes	Yes
Leaf Length	Yes	**No**	Yes	Yes	Yes	**No**	Yes	**No**
Leaf Width	Yes	Yes	Yes	**No**	Yes	Yes	Yes	**No**
Tassel Number	Yes	Yes	Yes	**No**	Yes	Yes	Yes	**No**
Ear Length	Yes	**No**	Yes	**No**	Yes	Yes	Yes	**No**

For the diploid plants:

Genotype (column)/ Phenotype (row)	A188/ O43--A188	A188/ O43 - - O43	A188/ B73--A188	A188/ B73--B73	A188/ W22--A188	A188/ W22--W22	O43/ A188--A188	O43/ A188--O43	B73/ A188--A188	B73/ A188--B73
4th Week Height	No	Yes	Yes	No	Yes	No	No	Yes	Yes	Yes
6th Week Height	No	Yes	Yes	No	No	Yes	Yes	Yes	Yes	Yes
Adult Height	Yes	Yes	Yes	Yes	Yes	No	Yes	Yes	Yes	Yes
Flower Time	Yes	Yes	Yes	Yes	Yes	Yes	Yes	Yes	Yes	Yes
Silk Time	Yes	Yes	Yes	Yes	Yes	Yes	Yes	Yes	Yes	Yes
Leaf Length	Yes	Yes	Yes	Yes	No	Yes	No	Yes	No	Yes
Leaf Width	Yes	Yes	Yes	Yes	Yes	Yes	Yes	No	Yes	Yes
Tassel Number	Yes	Yes	Yes	Yes	Yes	No	Yes	No	Yes	Yes
Ear Length	Yes	Yes	Yes	Yes	Yes	Yes	Yes	No	Yes	Yes

Genotype (column)/ Phenotype (row)	W22/ A188-A188	W22/ A188-W22	B73/ W22-B73	B73/ W22-W22	B73/ Oh43-B73	B73/ Oh43-Oh43	W22/ B73--B73	W22/ B73--W22	O43/ B73--B73	O43/ B73--O43
4th Week Height	Yes	Yes	No	Yes	Yes	No	No	Yes	No	Yes
6th Week Height	Yes	Yes	Yes	Yes	Yes	Yes	Yes	Yes	Yes	No
Adult Height	Yes	No	Yes	Yes	Yes	Yes	Yes	No	Yes	Yes
Flower Time	Yes	Yes	No	No	Yes	Yes	Yes	Yes	Yes	Yes
Silk Time	Yes	Yes	Yes	No	Yes	Yes	Yes	No	Yes	No
Leaf Length	Yes	Yes	Yes	Yes	Yes	Yes	Yes	Yes	Yes	Yes
Leaf Width	Yes	Yes	No	Yes	Yes	Yes	No	Yes	Yes	Yes
Tassel Number	Yes	No	Yes	Yes	No	No	Yes	Yes	Yes	Yes
Ear Length	Yes	Yes	Yes	Yes	Yes	Yes	No	Yes	No	No

Genotype (column)/ Phenotype (row)	O43/ W22-- Oh43	O43/ W22-- W22	W22/ Oh43-- Oh43	W22/ Oh43-- W22	A188/ O43 x B73/W22 --A188	A188/ O43 x B73/W22 -- O43	A188/ O43 x B73/W22 -- B73	A188/ O43 x B73/W22 -- W22
4th Week Height	**No**	**No**	Yes	Yes	**No**	**No**	Yes	**No**
6th Week Height	**No**	**No**	**No**	**No**	Yes	**No**	Yes	**No**
Adult Height	Yes	Yes	**No**	Yes	Yes	Yes	**No**	Yes
Flower Time	Yes	Yes	**No**	Yes	Yes	Yes	**No**	**No**
Silk Time	**No**	Yes	Yes	**No**	Yes	Yes	**No**	Yes
Leaf Length	Yes	**No**	Yes	Yes	**No**	Yes	Yes	Yes
Leaf Width	Yes	Yes	Yes	**No**	**No**	Yes	Yes	Yes
Tassel Number	Yes	**No**	Yes	Yes	Yes	Yes	Yes	Yes
Ear Length	Yes	Yes	**No**	**No**	Yes	Yes	**No**	Yes

Genotype (column)/ Phenotype (row)	B73/W22 x A188/O43 --A188	B73/W22 x A188/O43 -- O43	B73/W22 x A188/O43 -- B73	B73/W22 x A188/O43 -- W22
4th Week Height	Yes	Yes	Yes	Yes
6th Week Height	Yes	Yes	Yes	Yes
Adult Height	Yes	Yes	Yes	**No**
Flower Time	Yes	Yes	Yes	Yes
Silk Time	Yes	Yes	Yes	**No**
Leaf Length	Yes	**No**	Yes	Yes
Leaf Width	Yes	Yes	Yes	Yes
Tassel Number	Yes	Yes	Yes	**No**
Ear Length	Yes	Yes	**No**	**No**

REFERENCES

Faraway, J. J., 2006 Extending the Linear Model with R. Chapman & Hall, Boca Raton.

APPENDIX

SAS Code for the first question:

We use the following SAS code to the analysis for 4th week height phenotype:

```
proc mixed data=dep_rate_2n_4n_ploidy;
    class Field Generation Ploidy Genotype;
    model Height_at_4_Week = Generation Ploidy Genotype Generation*Ploidy
Generation*Genotype Ploidy*Genotype Generation*Ploidy*Genotype;
    lsmeans Generation*Ploidy*Genotype / slice=Generation*Genotype ;
    random Field;
 run;
```

We changed the phenotype in the above SAS code to do the analysis for all the phenotypes.

SAS Code for the second question:

We use the following SAS code to the analysis for 4th week height phenotype for the diploid plants:

```
proc mixed data=dep_rate_2n_ploidy;
    class Field Generation Genotype;
    model Height_at_4_Week = Generation Genotype Generation*Genotype;
    lsmeans Generation*Genotype / slice=Generation;
    random Field;
 run;
```

We changed the phenotype and used the data for both ploidies in the above SAS code to do the analysis for all the phenotypes and ploidies.

The SAS code for all the other questions used the logic of the above two questions.
