# Supplementary material for: Inbreeding Depression in Genotypically Matched Diploid and Tetraploid Maize
Source: Front Genet. 2020 Nov 30;11:564928. doi: 10.3389/fgene.2020.564928 (PMC7734256; doi:10.3389/fgene.2020.564928)
Supplement: Supplementary file 2 [file Data_Sheet_2.PDF]

# SUPPLEMENTARY MATERIAL #2

## 1. INTRODUCTION

The experiments to investigate inbreeding depression rates in diploid and tetraploid maize lines were conducted during 2008 and 2009 in Columbia, Missouri. The statistical analysis in this report is for the 2008 data.

The four diploid and tetraploid parental inbred maize lines A188 (2x, 4x), Oh43 (2x, 4x), B73 (2x, 4x) and W22 (2x, 4x) were used in this study. The following F1 hybrids from these parental lines were grown:
Oh43/A188 (2x), A188/Oh43 (2x), W22/B73 (2x), B73/W22 (2x), Oh43/W22 (2x), W22/Oh43 (2x), W22/A188 (2x), A188/W22 (2x), B73/A188 (2x), A188/B73 (2x), B73/Oh43 (2x), Oh43/B73 (2x), A188/Oh43 (2x) x B73/W22 (2x), B73/W22 (2x) x A188/Oh43 (2x), W22/B73 (4x), A188/Oh43 (4x), Oh43/A188/W22/B73 (4x).

Each F1 hybrid line was self mated for seven generations and progenies from generations 1, 3, 5, and 7 (named as S1, S3, S5, and S7) were used for data collection. Genetic segregation occurred after the first generation of the self mating population in this experiment. Kernels from three different S2 ears (resulting from self mating S1 plants) were used to produce the S3 to S7 lines, to account for the genetic diversity among the S1 plants derived from the same F1 hybrid. Thus, there were three selfing lineages for each genotype.

The experiments conducted in 2008 were based on a randomized complete block design. The maize lines from all ploidy levels, genotypes and generations were planted in two fields. Each maize line was grown in each of the two fields (blocks). Twenty seeds of maize lines were planted per row and, whenever possible, data from twelve plants were collected. The planting dates for the two blocks are as follows:

| Year 2008 |
| --- |
| **Block 2** – June 18th |
| **Block 3** – June 23rd |

Data on the following phenotypes were collected (the names in parentheses denote the variable names used in the analysis for the corresponding phenotype):

1. The number of days to anther emergence after planting. (flowering time)
2. The number of days to silk emergence after planting. (silk emergence time)
3. The ear length of the maize plant. (ear length)
4. The tassel branch number. (tassel branch number)
5. The height of the plant at $4^{th}$ week. ($4^{th}$ week height)
6. The height of the adult plant. (adult plant height)
7. The length of the $5^{th}$ leaf from the top. (length of the $5^{th}$ leaf from the top)
8. The width of the $5^{th}$ leaf from the top. (width of the $5^{th}$ leaf from the top)
9. The length of the $7^{th}$ leaf from the top. (length of the $7^{th}$ leaf from the top)
10. The width of the $7^{th}$ leaf from the top. (width of the $7^{th}$ leaf from the top)

The following biological questions about inbreeding depression rates are of interest:

1. Is the inbreeding depression rate different between diploid and tetraploid lines with the same genetic constitution?
2. Is the inbreeding depression rate different between lines with different genetic constitution, but the same ploidy level?
3. Does inbreeding depression occur in all phenotypes?
4. Is there depression in every diploid and tetraploid genotype?
5. How is the inbreeding depression rate affected by ploidy level, genetic constitution, and the interaction between ploidy level and genetic constitution?
6. Are there any parental effects on inbreeding depression rate?
7. Are the S7 lines different from their corresponding progenitor inbred lines?

The inbreeding depression rate for a phenotypic datum corresponding to the $m^{th}$ replicate from the $l^{th}$ field, in generation i, with genotype j, and ploidy k is defined as:

$$\text{Inbreeding Depression Rate}_{ijklm} = \frac{\text{Phenotype}_{ijklm}}{\sum_{m=1}^{12}\sum_{l=2}^{3}\text{Phenotype}_{1jklm} / 24} \tag{1}$$

For example, the inbreeding depression rate of ear length (phenotype) for any observation in Si generation of A188/Oh43 (2n) (genotype and ploidy) is defined/calculated as follows:

$$= \frac{\text{Observed Ear Length in } S_i \text{ from A188/Oh43 (2n)}}{\text{Mean of Ear Length in } F_1 \text{ from A188/Oh43 (2n)}}$$

The rest of this document is structured as follows: there is an Exploratory Data Analysis section that details the data visualization methods and plots for inbreeding depression rates for all the phenotypes. After that there is a Methodology section that details the statistical methods used in the analysis and it is followed by a Results section.

## 2. Exploratory Data Analysis

The density plot for all the phenotypes grouped according to field is in Figure 1. A density plot of a variable is an estimate of its probability density function based on the observed data under the assumption of random sampling from the population. In this particular case, the estimate of the unobservable probability density of a phenotype is plotted for the two different fields, separately. This figure allows us to check for the heterogeneity of data collected from different fields. The level of heterogeneity will be used to decide whether to plot the inbreeding depression rates of phenotypes based on different fields, or to pool the data from the fields.

**Figure 2.1:** Density plots for all the phenotypes colored according to the field. The density plots are for flowering time, silk emergence time, ear length, tassel branch number, $4^{th}$ week height, adult plant height, length of $5^{th}$ leaf from the top, width of the $5^{th}$ leaf from the top, length of $7^{th}$ leaf from the top, and width of the $7^{th}$ leaf from the top (left to right and top to bottom).

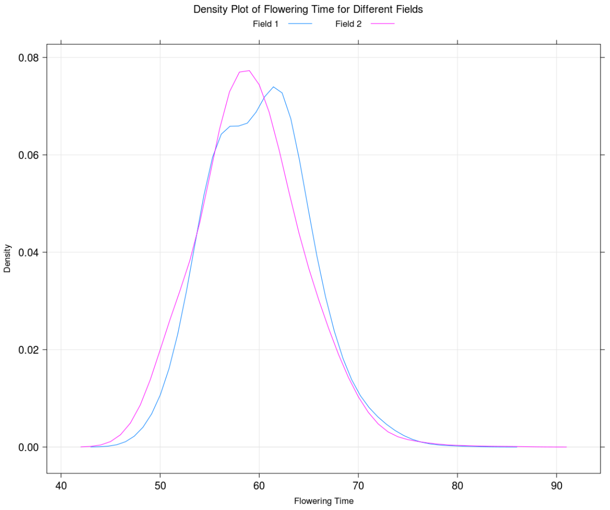
Density Plot of Flowering Time for Different Fields
Field 1
Field 2
Density
0.00
0.02
0.04
0.06
0.08
40
50
60
70
80
90
Flowering Time

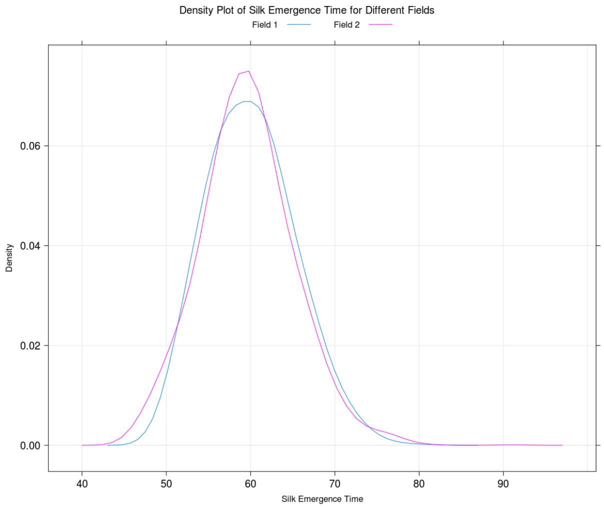
Density Plot of Silk Emergence Time for Different Fields
Field 1
Field 2
Density
0.00
0.02
0.04
0.06
40
50
60
70
80
90
Silk Emergence Time

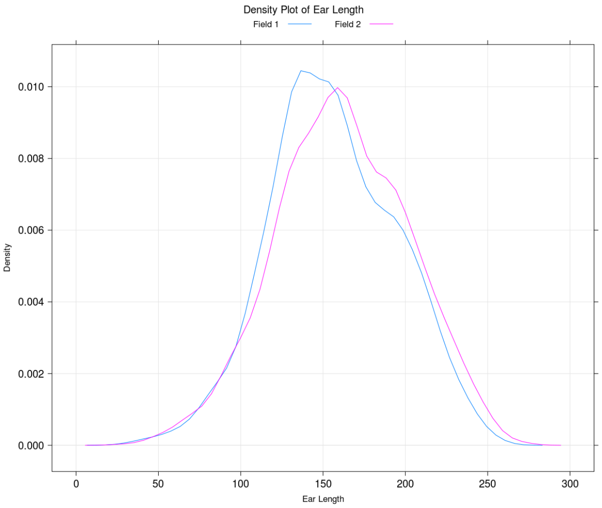
Density Plot of Ear Length
Field 1
Field 2
Density
0.000
0.002
0.004
0.006
0.008
0.010
0
50
100
150
200
250
300
Ear Length

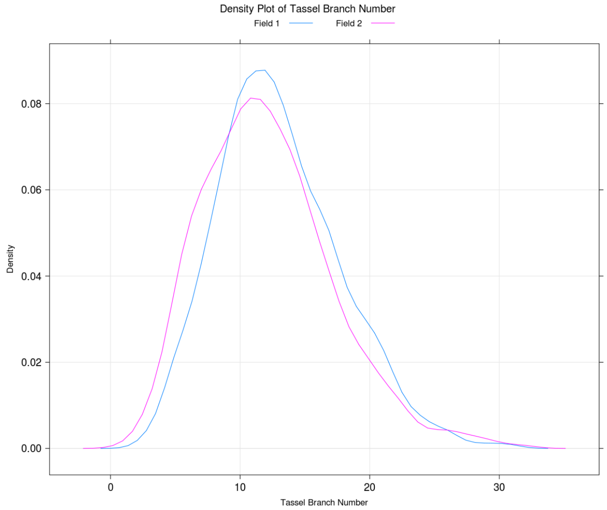
Density Plot of Tassel Branch Number
Field 1
Field 2
Density
0.00
0.02
0.04
0.06
0.08
0
10
20
30
Tassel Branch Number

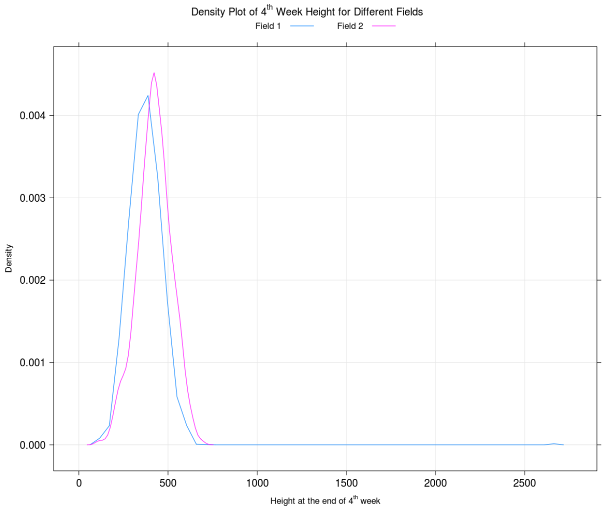
Density Plot of 4th Week Height for Different Fields
Field 1
Field 2
Density
0.000
0.001
0.002
0.003
0.004
0
500
1000
1500
2000
2500
Height at the end of 4th week

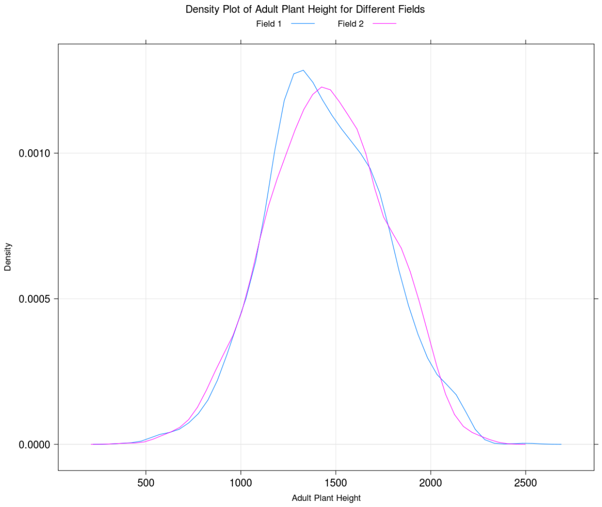
Density Plot of Adult Plant Height for Different Fields
Field 1
Field 2
Density
0.0000
0.0005
0.0010
500
1000
1500
2000
2500
Adult Plant Height

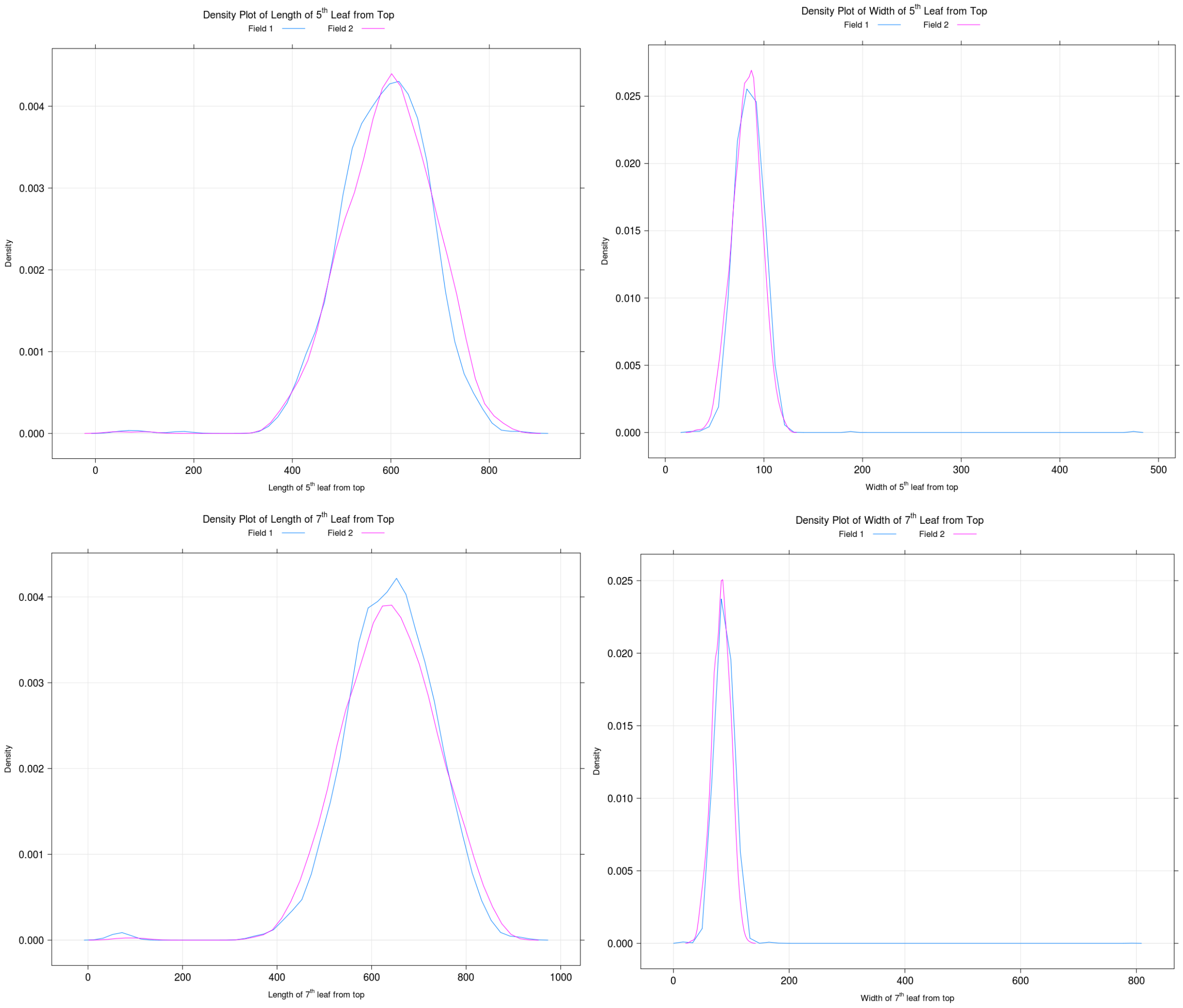


We start the data analysis by visualizing the inbreeding depression rates for all the phenotypes based on the five different generations F1, S1, S3, S5 and S7. The density plots in Figure 2.1 did not show any noticeable heterogeneity based on fields for all the phenotypic data, so we pooled the phenotypic data from the two fields for visualization purposes.

We also found that there was an outlier in the phenotypic data for the width of the 7$^{th}$ leaf from the top; it was replaced by the median of the phenotypic data to make the plots clearer and the statistical analysis (in the **Methodology** section) free from its influence.

The distribution of inbreeding depression rates for flowering time, silk emergence time, ear length, tassel branch number, $4^{th}$ week height, adult plant height, length of $5^{th}$ leaf from the top, width of the $5^{th}$ leaf from the top, length of $7^{th}$ leaf from the top, and width of the $7^{th}$ leaf from the top based on generation are summarized in Figures 2.2-2.11. The patterns in the inbreeding depression rates of the diploid plants and tetraploid plants are observed by looking at the red and blue box plots. The generation changes along the column from the F1 generation to S7 generation, from left to right along the columns. The plots for F1 generation are not interesting as the data from this generation was used to calculate the depression rates for the siblings. It is included to check the quality of data, as dividing the phenotypic data of F1 generation by its observed mean should lead to a depression rate for F1 very close to 1. The inter-quartile ranges of most of the box plots for the F1 generations include 1, indicating an overall good quality of the data. Below are the details of the plots based on the depression rate of a particular phenotype.

**The number of days to anther emergence after planting:** (Figure 2.2)
The depression rates for both ploidies **decrease** from genotype A188/Oh43 to W22/B73. The pattern for the decrease in the depression rates is strongest for the S5 and S7 generation. The box plots also show that the depression rate for the W22/B73 tetraploid plants is different from corresponding diploid plants, across all generations except S7.

**The number of days to silk emergence after planting:** (Figure 2.3)
The patterns in depression rates are similar to those observed in *flower time*.

**The ear length of the maize plant:** (Figure 2.4)
The depression rate **decreases** for the diploid and tetraploid plants and the pattern in tetraploid plants becomes stronger after generation S3. There is no clear difference between the depression rates of diploid and tetraploid plants across all generations.

**The tassel branch number:** (Figure 2.5)
The diploid plants have a **higher** depression rate than the tetraploid plants and the pattern is prominent in the Oh43/A188/W22/B73 genotype.

**The height of the plant at 4th week:** (Figure 2.6)

The inbreeding depression rate does not show any specific pattern across all ploidies or generations. In generations S1 and S3, the depression rate for the diploid plants is higher than the tetraploid plants and the diploid A188/Oh43 genotype has higher depression rate compared to the other two diploid genotypes.

**The height of the adult plant:** (Figure 2.7)

The depression rates for the diploid plants decrease from genotype A188/Oh43 to W22/B73 where as in the tetraploid plants Oh43/A188/W22/B73 genotype has the highest depression rate followed by A188/Oh43 and W22/B73 genotypes. This pattern is consistent across all generations.

**The length of the 5th leaf from the top:** (Figure 2.8)

The depression rate of the diploid plant **increases** across all generations from genotype A188/Oh43 to W22/B73. The depression rate for tetraploid B73/W22 genotype is **lower** than that of the diploid genotype; the pattern is strong after the S3 generation.

**The width of the 5th leaf from the top:** (Figure 2.9)

There are **no visible differences** between the depression rates of the diploid and the tetraploid plants. But we observe that the depression rate for the tetraploid Oh43/A188/W22/B73 genotype is higher compared to the other tetraploid genotypes, specifically in generations S1, S3 and S5.

**The length of the 7th leaf from the top:** (Figure 2.10)

There are **no visible differences** between the depression rates of the diploid and the tetraploid plants across all generations and genotypes. But we observe that the depression rate for the diploid Oh43/A188/W22/B73 genotype is lower compared to the other diploid genotypes across all generations.

**The width of the 7th leaf from the top:** (Figure 2.11)

There are **no visible differences** between the depression rates of the diploid and the tetraploid plants across all generations and genotypes. But we observe that the depression rate for the tetraploid Oh43/A188/W22/B73 genotype is higher compared to the other tetraploid genotypes, specifically in generations S1, S3 and S5.

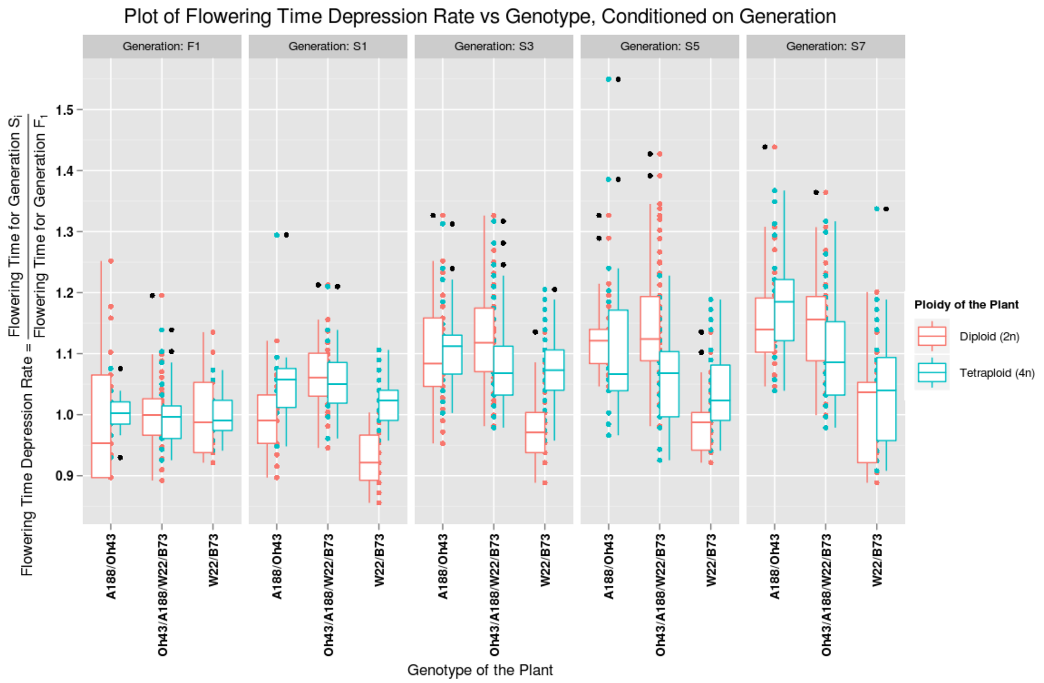


**Figure 2.2**: Plot of depression rate for flowering time vs the genotype of the plant, conditioned on generations F1, S1, S3, S5 and S7. The data for diploid plants are graphed in red and the tetraploid plants are graphed in blue. The patterns for a particular ploidy can be observed by looking at the box plots of the corresponding color. The generation varies from F1 to S7 column-wise.

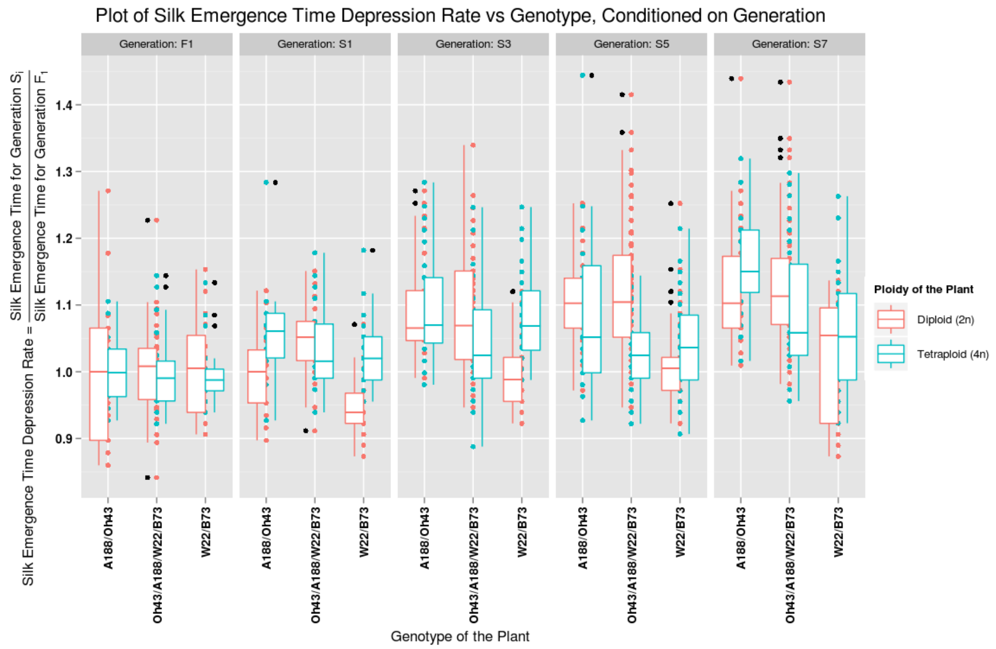


**Figure 2.3**: Plot of depression rate for silk time vs the genotype of the plant, conditioned on generations F1, S1, S3, S5 and S7. The data for diploid plants are graphed in red and the tetraploid plants are graphed in blue. The patterns for a particular ploidy can be observed by looking at the box plots of the corresponding color. The generation varies from F1 to S7 column-wise.

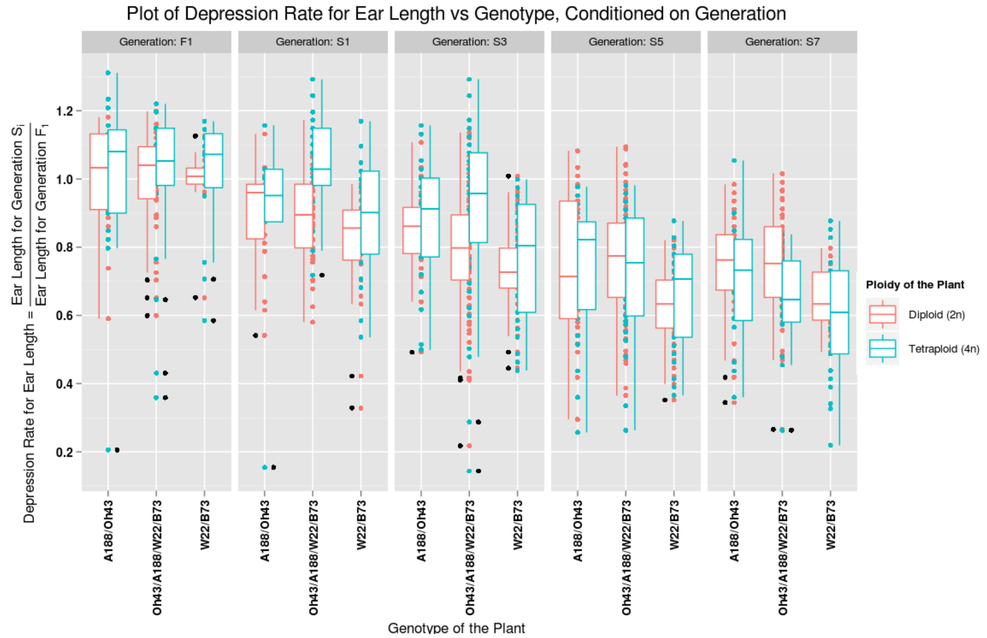


**Figure 2.4:** Plot of depression rate for ear length vs the genotype of the plant, conditioned on generations F1, S1, S3, S5 and S7. The data for diploid plants are graphed in red and the tetraploid plants are graphed in blue. The patterns for a particular ploidy can be observed by looking at the box plots of the corresponding color. The generation varies from F1 to S7 column-wise.

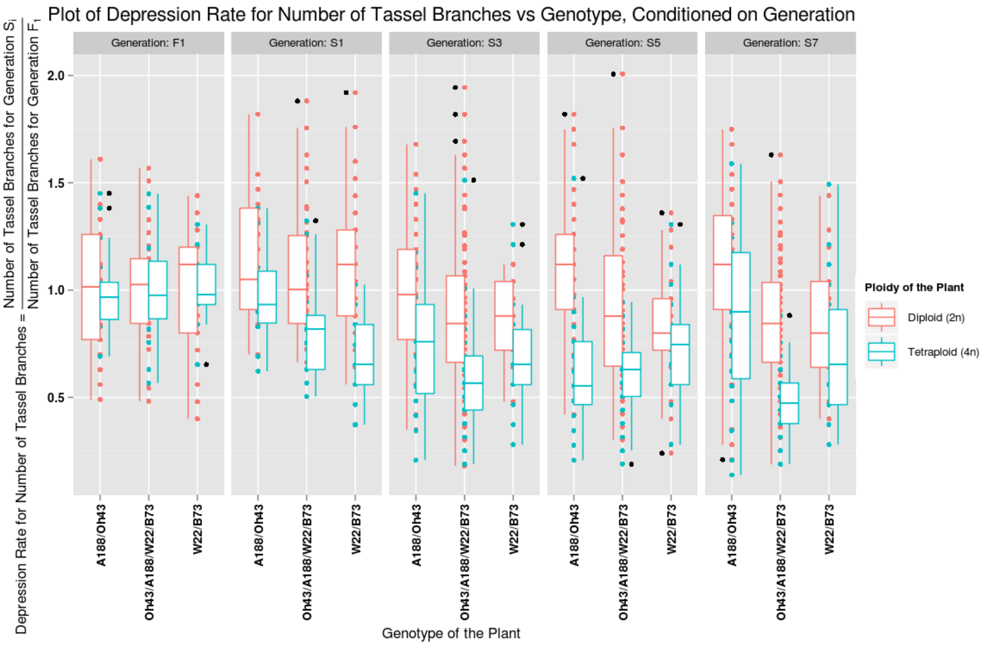


**Figure 2.5:** Plot of depression rate for the number of tassel branches vs the genotype of the plant, conditioned on generations F1, S1, S3, S5 and S7. The data for diploid plants are graphed in red and the tetraploid plants are graphed in blue. The patterns for a particular ploidy can be observed by looking at the box plots of the corresponding color. The generation varies from F1 to S7 column-wise.

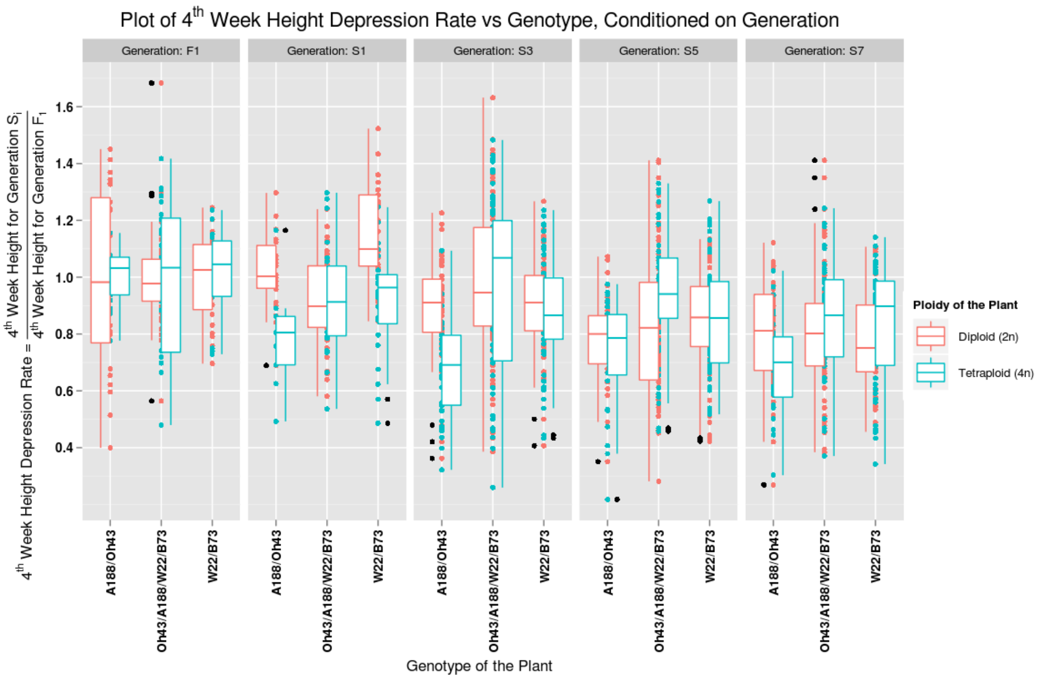


**Figure 2.6**: Plot of depression rate for $4^{th}$ week height vs the genotype of the plant, conditioned on generations F1, S1, S3, S5 and S7. The data for diploid plants are graphed in red and the tetraploid plants are graphed in blue. The patterns for a particular ploidy can be observed by looking at the box plots of the corresponding color. The generation varies from F1 to S7 column-wise.

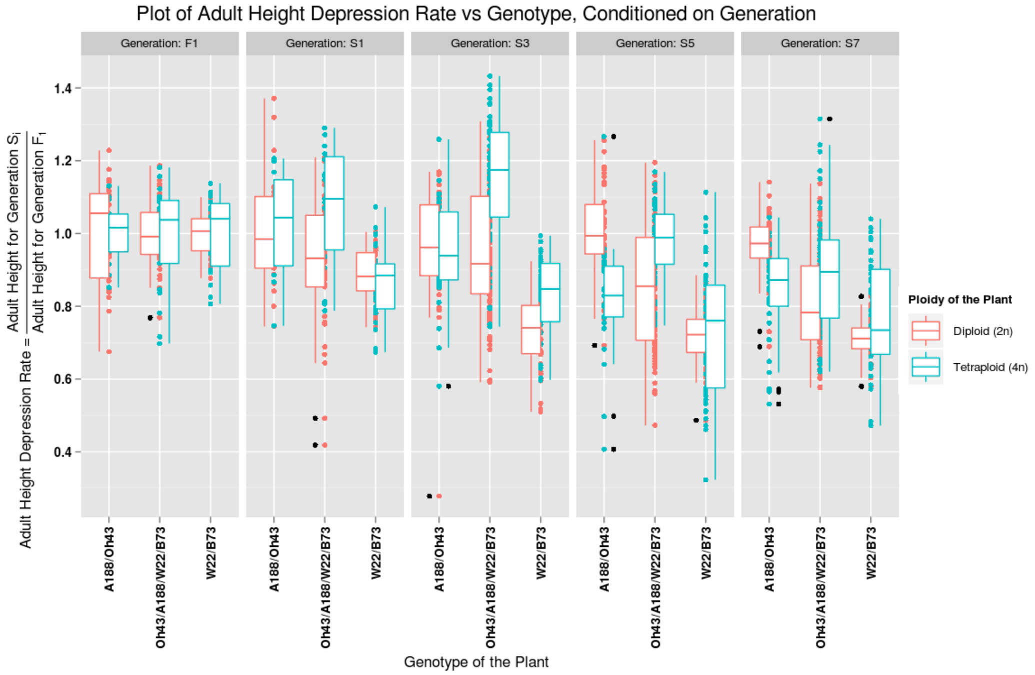


**Figure 2.7:** Plot of depression rate for adult height vs the genotype of the plant, conditioned on generations F1, S1, S3, S5 and S7. The data for diploid plants are graphed in red and the tetraploid plants are graphed in blue. The patterns for a particular ploidy can be observed by looking at the box plots of the corresponding color. The generation varies from F1 to S7 column-wise.

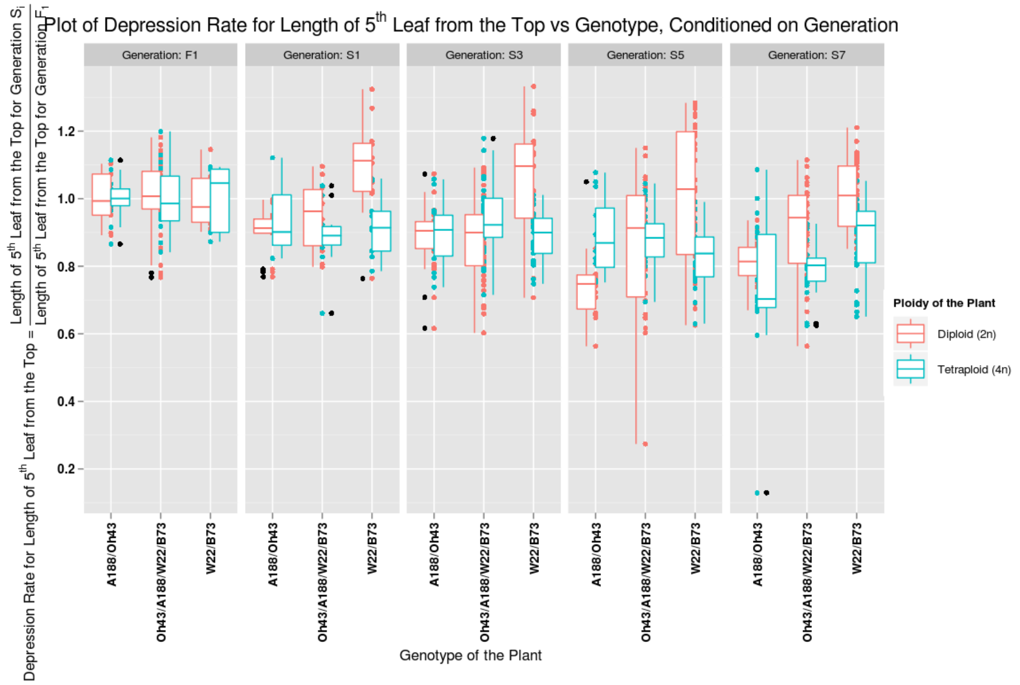


**Figure 2.8:** Plot of depression rate for length of 5$^{th}$ leaf vs the genotype of the plant, conditioned on generations F1, S1, S3, S5 and S7. The data for diploid plants are graphed in red and the tetraploid plants are graphed in blue. The patterns for a particular ploidy can be observed by looking at the box plots of the corresponding color. The generation varies from F1 to S7 column- wise.

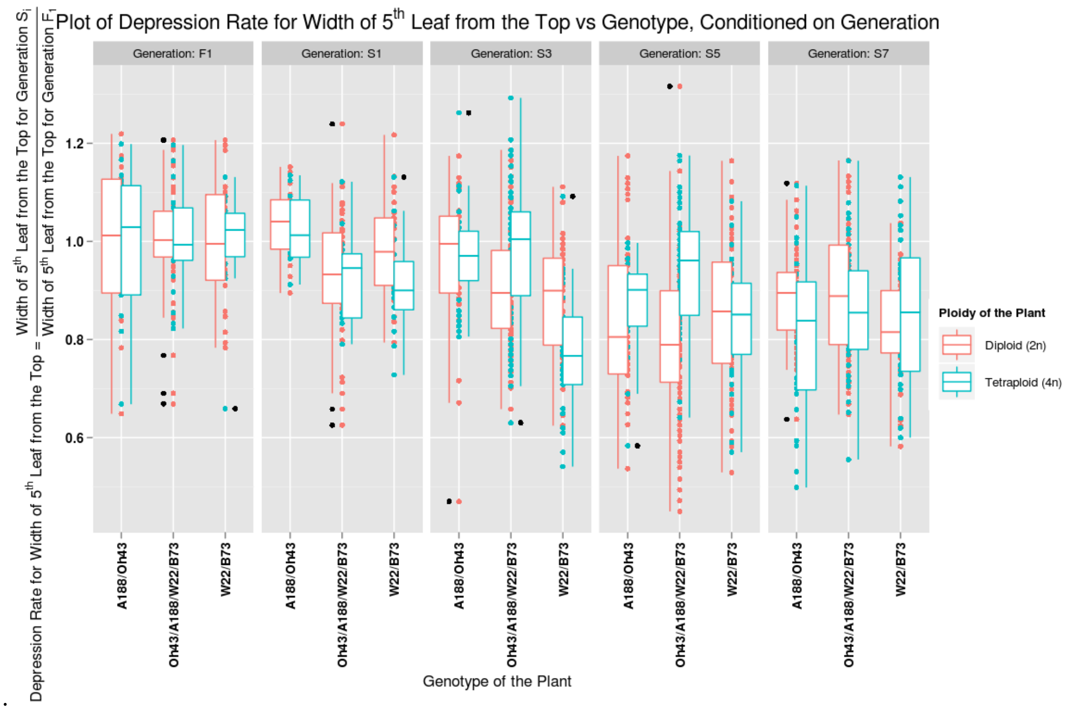


**Figure 2.9:** Plot of depression rate for width of 5th leaf vs the genotype of the plant, conditioned on generations F1, S1, S3, S5 and S7. The data for diploid plants are graphed in red and the tetraploid plants are graphed in blue. The patterns for a particular ploidy can be observed by looking at the box plots of the corresponding color. The generation varies from F1 to S7 column-wise.

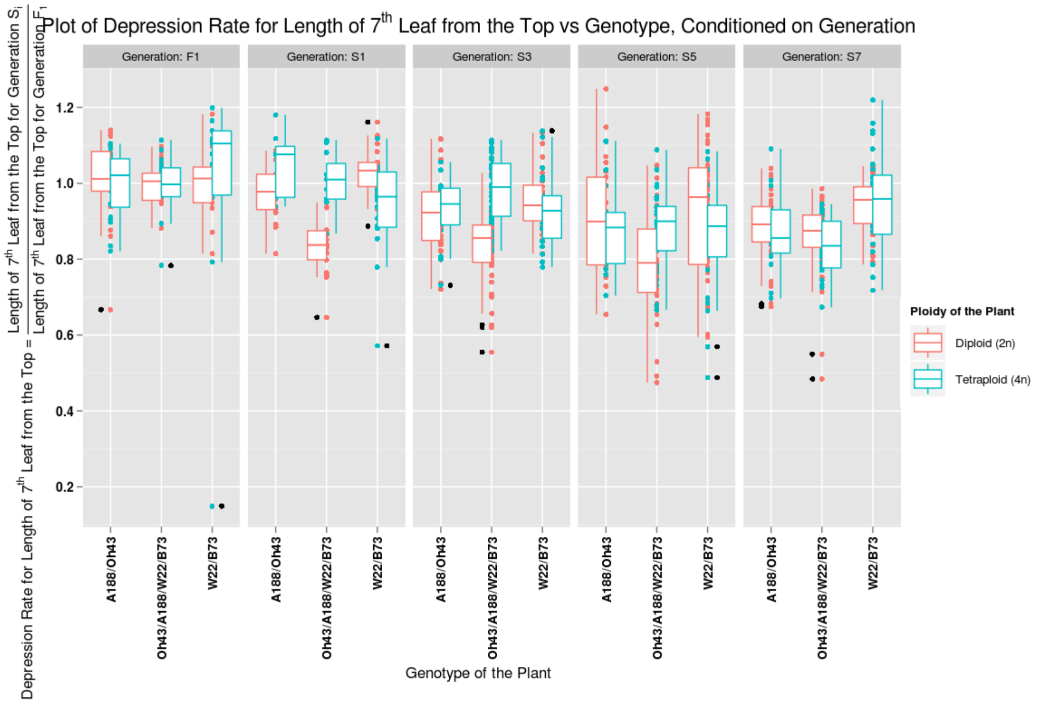


**Figure 2.10:** Plot of depression rate for length of 5th leaf vs the genotype of the plant, conditioned on generations F1, S1, S3, S5 and S7. The data for diploid plants are graphed in red and the tetraploid plants are graphed in blue. The patterns for a particular ploidy can be observed by looking at the box plots of the corresponding color. The generation varies from F1 to S7 column-wise.

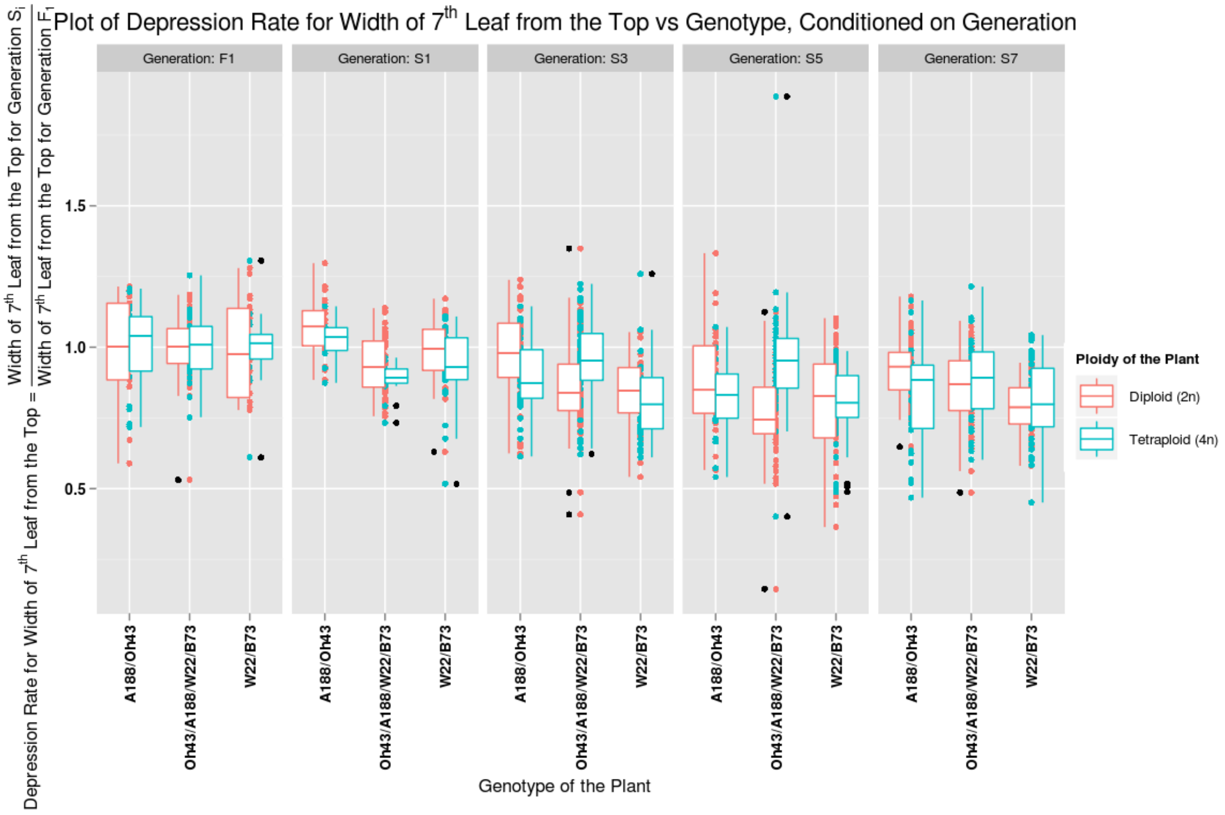


**Figure 2.11:** Plot of depression rate for width of 7th leaf vs the genotype of the plant, conditioned on generations F1, S1, S3, S5 and S7. The data for diploid plants are graphed in red and the tetraploid plants are graphed in blue. The patterns for a particular ploidy can be observed by looking at the box plots of the corresponding color. The generation varies from F1 to S7 column-wise.

## 3. Methodology – Analysis of Variance (ANOVA)

The analyses to answer the questions *1 –7* in **Section 1** are similar to the methods mentioned in the *Section 3 (Methodology)* of the *2009 Report*. The only changes in the model for this report are the number of fields and the phenotypes. The number of fields in this report is two, namely: 2 and 3. There are no data for 6th week height phenotype and there are two additional phenotypes analyzed in this report, namely: the length of 7th leaf from top and the width of 7th leaf from top. The model details have been omitted from this report; for details about the model formulation, please refer to the *2009 Report*.

## 4. RESULTS

The summary of the results for all the questions are as follows. In all the summaries below *Yes* denotes that the null hypothesis was rejected in favor of the alternative and ***No*** denotes that there was not enough evidence to reject the null hypothesis.

1. **Is the inbreeding depression rate different between diploid and tetraploid lines with the same genetic constitution?**

We assumed that the tetraploid Oh43/A188/W22/B73 genotype was identical to A188/Oh43 x B73/W22 and B73/W22 x A188/Oh43 diploid genotypes. In the table below, in a particular phenotypic column, *Yes* denotes that the null hypothesis was rejected in favor of the alternative and ***No*** denotes that there was not enough evidence to reject the null hypothesis for that phenotype (see: *2009 Report* **- Section 3, H1**).

| Generation | Genotype (Maize Lines) | Flower-ing time | Silk Time | No. of Tassel Braches | Ear Length | Height at the end of 4 weeks | Adult Height | Length of the 5th leaf | Width of the 5th leaf | Length of the 7th leaf | Width of the 7th leaf |
|---|---|---|---|---|---|---|---|---|---|---|---|
| | | | | | | | | | | | |
| F1 | A188/Oh43 | **No** | **No** | **No** | **No** | **No** | **No** | **No** | **No** | **No** | **No** |
| F1 | Oh43/A188/W22/B73 | **No** | **No** | **No** | **No** | **No** | **No** | **No** | **No** | **No** | **No** |
| F1 | W22/ B73 | **No** | **No** | **No** | **No** | **No** | **No** | **No** | **No** | **No** | **No** |
| S1 | A188/Oh43 | Yes | Yes | **No** | **No** | Yes | **No** | **No** | **No** | **No** | **No** |
| S1 | Oh43/A188/W22/B73 | **No** | **No** | Yes | Yes | **No** | Yes | **No** | **No** | Yes | **No** |
| S1 | W22/ B73 | Yes | Yes | Yes | **No** | Yes | **No** | Yes | Yes | Yes | **No** |
| S3 | A188/Oh43 | **No** | **No** | Yes | **No** | Yes | **No** | **No** | **No** | **No** | Yes |
| S3 | Oh43/A188/W22/B73 | Yes | Yes | Yes | Yes | **No** | Yes | Yes | Yes | Yes | Yes |
| S3 | W22/ B73 | Yes | Yes | Yes | **No** | **No** | **Yes** | Yes | Yes | **No** | **No** |
| S5 | A188/Oh43 | **No** | **No** | Yes | **No** | **No** | Yes | Yes | **No** | **No** | Yes |
| S5 | Oh43/A188/W22/B73 | Yes | Yes | Yes | **No** | Yes | Yes | **No** | Yes | Yes | Yes |
| S5 | W22/ B73 | Yes | **No** | **No** | **No** | **No** | **No** | Yes | **No** | Yes | **No** |
| S7 | A188/Oh43 | **No** | Yes | Yes | **No** | Yes | Yes | **No** | Yes | **No** | Yes |
| S7 | Oh43/A188/W22/B73 | Yes | Yes | Yes | Yes | **No** | Yes | Yes | **No** | **No** | **No** |
| S7 | W22/ B73 | Yes | Yes | **No** | **No** | **No** | Yes | Yes | **No** | **No** | **No** |

2. **Is the inbreeding depression rate different between lines with different genetic constitution but the same ploidy?**

All the genotypic effects of diploid lines were significant. In the tetraploid lines all the genotypic effects were significant with the following exceptions:

**The number of days to anther emergence after planting:**

The genotypic effect was not significant in the S1 and S3 generations.

**The ear length of the maize plant:**

The overall interaction between genotype and generation was not significant. The genotypic effect was not significant in the S5 and S7 generations.

**The height of the plant at 4th week:**

The genotypic effect was not significant in the S1 generation.

**The length of the 5th leaf from the top:**

The overall genotypic effect was not significant as well as it was not significant S1, S3 and S5 generations.

**The width of the 5th leaf from the top:**

The genotypic effect was not significant in the S1 and S7 generations.

**The length of the 7th leaf from the top:**

The overall genotypic effect was not significant as well as it was not significant S1, S3 and S5 generations.

3. **Does inbreeding depression occur in all the measured phenotypes?**

Inbreeding depression occur in all the measured phenotypes. The results for a particular generation and genotype are summarized in the results of first question for all the phenotypes. The results for a particular generation and ploidy are summarized in the results of second question for all the phenotypes.

4. **Is there depression in every diploid and tetraploid genotype?**

There is inbreeding depression in all the genotypes and in both the ploidies across all siblings. The results are summarized in the analysis of the first and second questions.

5. **How is the inbreeding depression rate affected by ploidy, genetic constitution and the interaction between ploidy and genetic constitution?**

Inbreeding depression rate is affected by ploidy, genetic constitution and the interaction between ploidy and genetic constitution.

6. **Are there any parental effects on inbreeding depression rate?**

Following table summarizes the results of statistical hypotheses tests (*2009 Report* **- Section 3, H6**) to determine the difference of inbreeding depression rate between reciprocal pairs based on generations F1, S1, S3, S5 and S7 and for all the phenotypes. For a particular phenotype, in the table below, row represents the generation and the columns denote one of the seven reciprocal crosses. *Yes* denotes that the null hypothesis was rejected in favor of the alternative and ***No*** denotes that there was not enough evidence to reject the null hypothesis (*2009 Report* **- Section 3, H6**). NA denotes that there were not enough data to perform the hypotheses test.

| **Generation** | **AB** | **AO** | **AW** | **ABOW** | **BO** | **BW** | **OW** |
|---|---|---|---|---|---|---|---|
| **Number of Days to Emergence of Anthers** | | | | | | | |
| **F1** | **No** | **No** | **No** | **No** | **No** | **No** | **No** |
| **S1** | **No** | **No** | **No** | Yes | Yes | Yes | Yes |
| **S3** | Yes | **No** | Yes | Yes | **No** | Yes | **No** |
| **S5** | Yes | Yes | Yes | Yes | **No** | Yes | Yes |
| **S7** | Yes | Yes | Yes | Yes | Yes | Yes | **No** |
| | | | | | | | |
| **Number of Days to Emergence of Silk** | | | | | | | |

| | | | | | | | |
|---|---|---|---|---|---|---|---|
| **F1** | **No** | **No** | **No** | **No** | **No** | **No** | **No** |
| **S1** | No | **No** | Yes | Yes | **No** | Yes | Yes |
| **S3** | Yes | **No** | Yes | Yes | Yes | Yes | Yes |
| **S5** | Yes | **No** | Yes | Yes | Yes | Yes | Yes |
| **S7** | Yes | **No** | Yes | Yes | Yes | Yes | **No** |
| | | | | | | | |
| **Ear Length** | | | | | | | |
| **F1** | **No** | **No** | **No** | **No** | **No** | **No** | **No** |
| **S1** | **No** | **No** | Yes | **No** | Yes | Yes | **No** |
| **S3** | **No** | Yes | **No** | Yes | **No** | Yes | Yes |
| **S5** | **Yes** | **No** | **No** | **No** | Yes | Yes | Yes |
| **S7** | **No** | Yes | **No** | Yes | **No** | **No** | Yes |
| | | | | | | | |
| **Number of Tassel Branches** | | | | | | | |
| **F1** | **No** | **No** | **No** | **No** | **No** | **No** | **No** |
| **S1** | **No** | **No** | **No** | Yes | **No** | Yes | **No** |
| **S3** | Yes | Yes | Yes | Yes | Yes | Yes | **No** |
| **S5** | **No** | Yes | Yes | Yes | Yes | **No** | Yes |
| **S7** | Yes | Yes | Yes | Yes | Yes | Yes | Yes |
| | | | | | | | |
| **Height at the end of $4^{th}$ Week** | | | | | | | |
| **F1** | **No** | **No** | **No** | **No** | **No** | **No** | **No** |
| **S1** | **No** | Yes | Yes | **No** | **No** | Yes | Yes |
| **S3** | Yes | Yes | Yes | Yes | Yes | Yes | **No** |
| **S5** | **No** | Yes | Yes | Yes | **No** | **No** | Yes |
| **S7** | **No** | Yes | Yes | Yes | Yes | **No** | Yes |
| | | | | | | | |
| **Adult Plant Height** | | | | | | | |
| **F1** | **No** | **No** | **No** | **No** | **No** | **No** | **No** |
| **S1** | Yes | **No** | **No** | Yes | **No** | **No** | Yes |

| | | | | | | | |
|---|---|---|---|---|---|---|---|
| **S3** | Yes | Yes | Yes | Yes | Yes | **No** | Yes |
| **S5** | Yes | Yes | Yes | Yes | Yes | Yes | Yes |
| **S7** | Yes | Yes | Yes | Yes | Yes | Yes | Yes |
| | | | | | | | |
| **Length of the 5th Leaf from the Top** | | | | | | | |
| **F1** | **No** | **No** | **No** | **No** | **No** | **No** | **No** |
| **S1** | Yes | Yes | **No** | NA | **No** | **No** | **No** |
| **S3** | **No** | **No** | **No** | **No** | Yes | Yes | Yes |
| **S5** | **No** | Yes | **No** | **No** | Yes | Yes | Yes |
| **S7** | **No** | **No** | **No** | **No** | **No** | Yes | Yes |
| | | | | | | | |
| **Width of the 5th Leaf from the Top** | | | | | | | |
| **F1** | **No** | **No** | **No** | **No** | **No** | **No** | **No** |
| **S1** | **No** | **No** | **No** | Yes | **No** | Yes | **No** |
| **S3** | **No** | Yes | Yes | Yes | Yes | Yes | Yes |
| **S5** | Yes | **No** | **No** | Yes | **No** | **No** | Yes |
| **S7** | **No** | **No** | **No** | Yes | Yes | Yes | Yes |
| | | | | | | | |
| **Length of the 7th Leaf from the Top** | | | | | | | |
| **F1** | **No** | **No** | **No** | **No** | **No** | **No** | **No** |
| **S1** | Yes | **No** | **No** | **No** | **No** | Yes | Yes |
| **S3** | **No** | **No** | **No** | **No** | Yes | Yes | Yes |
| **S5** | **No** | Yes | Yes | **No** | Yes | Yes | Yes |
| **S7** | **No** | Yes | Yes | Yes | Yes | Yes | Yes |
| | | | | | | | |
| **Width of the 7th Leaf from the Top** | | | | | | | |
| **F1** | **No** | **No** | **No** | **No** | **No** | **No** | **No** |
| **S1** | **No** | Yes | **No** | **No** | **No** | **No** | **No** |
| **S3** | **No** | Yes | Yes | Yes | Yes | **No** | Yes |
| **S5** | **No** | Yes | **No** | Yes | **No** | **No** | Yes |

| S7 | Yes | Yes | No | Yes | No | Yes | Yes |
|---|---|---|---|---|---|---|---|
| | | | | | | | |

7. **Are the S7 lines different from their corresponding progenitor inbred lines?**

Following table summarizes the results of statistical hypotheses tests (*2009 Report* **- Section 3, H7**) to determine the difference of phenotypes between the S7 generation and corresponding inbred lines for the diploid and tetraploid plants. In the table below, row represents the phenotype and the columns denote S7 and one of its parents for all the genotypes. There are separate tables for tetraploid and diploid plants as the genotypes differ between the two ploidies. The column labels have the naming scheme: S7 genotype -- Parent genotype. NA denotes that there weren't enough data to perform the hypotheses test.

**For the tetraploid plants:**

| Genotype (column)/ Phenotype (row) | A188/O43-- A188 | A188/ O43 - - O43 | W22/ B73-- B73 | W22/ B73-- W22 | O43/ A188/ W22/B73 --A188 | O43/ A188/ W22/B73 -- O43 | O43/ A188/ W22/B73 -- B73 | O43/ A188/ W22/B73 -- W22 |
|---|---|---|---|---|---|---|---|---|
| Flower Time | No | Yes | No | Yes | Yes | Yes | Yes | Yes |
| Silk Time | No | NA | No | Yes | No | NA | Yes | Yes |
| Ear Length | Yes | NA | Yes | No | No | NA | Yes | No |
| Tassel Number | No | Yes | No | Yes | Yes | Yes | No | Yes |
| 4th Week Height | No | Yes | No | No | Yes | Yes | No | No |
| Adult Height | Yes | No | No | Yes | Yes | Yes | No | Yes |
| 5th Leaf Length | No | No | No | No | Yes | No | Yes | No |
| 5 th Leaf Width | No | No | No | No | Yes | No | No | No |
| 7th Leaf Length | Yes | No | No | Yes | Yes | Yes | Yes | Yes |
| 7th Leaf Width | Yes | No | Yes | Yes | Yes | No | Yes | Yes |

**For the diploid plants:**

| Genotype (column)/ Phenotype (row) | A188/ O43-- A188 | A188/ O43 - - O43 | A188/ B73-- A188 | A188/ B73-- B73 | A188/ W22-- A188 | A188/ W22-- W22 | O43/ A188-- A188 | O43/ A188-- O43 | B73/ A188-- A188 | B73/ A188-- B73 |
|---|---|---|---|---|---|---|---|---|---|---|
| Flower Time | Yes | **No** | Yes | Yes | Yes | Yes | Yes | Yes | Yes | Yes |
| Silk Time | Yes | **No** | Yes | Yes | Yes | Yes | Yes | Yes | Yes | Yes |
| Ear Length | Yes | Yes | **No** | Yes | **No** | Yes | Yes | Yes | **No** | Yes |
| Tassel Number | Yes | Yes | Yes | Yes | Yes | **No** | Yes | Yes | Yes | Yes |
| 4th Week Height | **No** | **No** | Yes | **No** | **No** | **No** | **No** | **No** | **No** | **No** |
| Adult Height | Yes | **No** | Yes | Yes | Yes | **No** | Yes | Yes | Yes | Yes |
| 5th Leaf Length | **No** | **No** | Yes | **No** | **No** | **No** | **No** | Yes | **No** | **No** |
| 5 th Leaf Width | Yes | Yes | Yes | **No** | Yes | Yes | Yes | Yes | **No** | Yes |
| 7th Leaf Length | **No** | **No** | Yes | Yes | **No** | **No** | **No** | Yes | **No** | Yes |
| 7th Leaf Width | Yes | Yes | Yes | **No** | Yes | Yes | Yes | Yes | Yes | **No** |

| Genotype (column)/ Phenotype (row) | W22/ A188- A188 | W22/ A188- W22 | B73/ W22- B73 | B73/ W22- W22 | B73/ Oh43- B73 | B73/ Oh43- Oh43 | W22/ B73-- B73 | W22/ B73-- W22 | O43/ B73-- B73 | O43/ B73-- O43 |
|---|---|---|---|---|---|---|---|---|---|---|
| Flower Time | Yes | **No** | Yes | Yes | **No** | Yes | Yes | **No** | **No** | Yes |
| Silk Time | Yes | Yes | Yes | **No** | Yes | Yes | **No** | **No** | **No** | Yes |
| Ear Length | **No** | Yes | Yes | Yes | **No** | Yes | Yes | **No** | **No** | Yes |
| Tassel Number | Yes | **No** | **No** | Yes | **No** | **No** | Yes | Yes | Yes | Yes |
| 4th Week Height | **No** | **No** | Yes | **No** | **No** | **No** | **No** | **No** | **No** | Yes |
| Adult Height | Yes | **No** | Yes | Yes | **No** | Yes | Yes | Yes | Yes | **No** |
| 5th Leaf Length | Yes | **No** | **No** | **No** | **No** | **No** | **No** | Yes | **No** | **No** |
| 5 th Leaf Width | Yes | **No** | **No** | Yes | Yes | **No** | **No** | Yes | **No** | Yes |
| 7th Leaf Length | **No** | Yes | **No** | **No** | **No** | **No** | **No** | Yes | Yes | **No** |
| 7th Leaf Width | Yes | Yes | Yes | **No** | Yes | **No** | Yes | Yes | Yes | Yes |

| Genotype (column)/ Phenotype (row) | O43/ W22-- Oh43 | O43/ W22-- W22 | W22/ Oh43-- Oh43 | W22/ Oh43-- W22 | A188/ O43 x B73/W22 --A188 | A188/ O43 x B73/W22 -- O43 | A188/ O43 x B73/W22 -- B73 | A188/ O43 x B73/W22 -- W22 |
|---|---|---|---|---|---|---|---|---|
| Flower Time | Yes | Yes | **No** | **No** | Yes | **No** | Yes | **No** |
| Silk Time | Yes | Yes | **No** | **No** | Yes | **No** | **No** | **No** |
| Ear Length | Yes | Yes | Yes | **No** | Yes | Yes | **No** | **No** |
| Tassel Number | Yes | Yes | Yes | Yes | Yes | Yes | Yes | **No** |
| 4th Week Height | **No** | **No** | **No** | **No** | **No** | **No** | **No** | **No** |
| Adult Height | **No** | Yes | Yes | Yes | Yes | Yes | **No** | Yes |
| 5th Leaf Length | **No** | **No** | **No** | **No** | **No** | **No** | **No** | **No** |
| 5 th Leaf Width | **No** | **No** | Yes | **No** | Yes | Yes | **No** | Yes |
| 7th Leaf Length | **No** | **No** | Yes | Yes | Yes | **No** | **No** | **No** |
| 7th Leaf Width | **No** | **No** | **No** | **No** | Yes | Yes | **No** | Yes |

| Genotype (column)/ Phenotype (row) | B73/W22 x A188/O43 --A188 | B73/W22 x A188/O43 -- O43 | B73/W22 x A188/O43 -- B73 | B73/W22 x A188/O43 -- W22 |
|---|---|---|---|---|
| Flower Time | Yes | Yes | Yes | **No** |
| Silk Time | Yes | **No** | Yes | **No** |
| Ear Length | Yes | Yes | **No** | **No** |
| Tassel Number | Yes | Yes | Yes | Yes |
| 4th Week Height | **No** | **No** | **No** | **No** |
| Adult Height | Yes | **No** | Yes | Yes |
| 5th Leaf Length | Yes | Yes | **No** | Yes |
| 5 th Leaf Width | Yes | Yes | Yes | **No** |
| 7th Leaf Length | Yes | Yes | **No** | Yes |
| 7th Leaf Width | Yes | **No** | Yes | **No** |
