# Supplementary material for: Inbreeding Depression in Genotypically Matched Diploid and Tetraploid Maize
Source: Front Genet. 2020 Nov 30;11:564928. doi: 10.3389/fgene.2020.564928 (PMC7734256; doi:10.3389/fgene.2020.564928)
Supplement: Supplementary file 3 [file Data_Sheet_3.PDF]

SUPPLEMENTARY MATERIAL #3

INBREEDING DEPRESSION RATES IN DIPLOID AND TETRAPLOID MAIZE LINES YEAR 2008 AND 2009

1. INTRODUCTION

The experiments to investigate inbreeding depression rates in diploid and tetraploid maize lines were conducted during 2008 and 2009 in Columbia, Missouri. This report summarizes the analysis of the seven questions investigated in the manuscript.

The phenotypic traits were measured in several number of biological replicates in three fields in the years 2008 and 2009. Due to unforeseen reasons, data from field 1 could not be collected in 2008. The following phenotypic data were collected in 2008 and 2009 on the following phenotypes:

2008:

1. The number of days to anther emergence after planting.
2. The number of days to silk emergence after planting.
3. The ear length of the maize plant.
4. The tassel branch number.
5. The height of the plant at 4th week.
6. The height of the adult plant.
7. The length of the 5th leaf from the top.
8. The width of the 5th leaf from the top.
9. The length of the 7th leaf from the top.
10. The width of the 7th leaf from the top.

2009:

1. The number of days to anther emergence after planting.
2. The number of days to silk emergence after planting.
3. The ear length of the maize plant.

4. The tassel branch number.
5. The height of the plant at 4th week.
6. The height of the plant at 6th week.
7. The height of the adult plant.
8. The length of the 5th leaf from the top.
9. The width of the 5th leaf from the top.

Let y_{ijklm} denote the phenotypic data for the *m*th replicate from the *l*th field, in generation i, with genotype j, and ploidy k in a particular year. Then, we performed the following analysis:

- We have summarized the data for every field *l*th field, in generation i, with genotype j, and ploidy k in a year by averaging over the biological replicates; that is, we use $\sum_{m=1}^{M} y_{ijklm} / M$ as the observed data, where M is the number of biological replicates and equals 12 in most cases.
- We have labeled the generations $i = F0, S1, S3, S5, S7$ as 0, 1, 3, 5, and 7. The presence of inbreeding depression is indicated by the decreasing values of $\sum_{m=1}^{M} y_{ijklm} / M$ on an average as i increases from 0 to 7.
- The summarized data from the different fields and years are analyzed separately.

The remainder of this document is structured as follows: Section 2 describes the statistical analysis for the seven questions considered in the manuscript; Section 3 summarizes the results; and an Appendix contains details about organization of results.

2. Statistical Methodology

We now discuss hypothesis tests that are used to answer the seven questions considered in the manuscript.

1. Is the inbreeding depression rate different between diploid and tetraploid lines with the same genetic constitution?

There are three genetic constitutions that have diploid and tetraploid plants in the 2008 and 2009 data. They include "Oh43/A188/W22/B73", "A188/Oh43", and "W22/B73" genotypes. Let y be the observed value of a phenotypic trait, $ploidy$ be 0 and 1 for diploid and tetraploid plants, and gen be 0, 1, 3, 5, and 7 depending on the generation of the plant. Then, a linear regression model of y on $ploidy$ and gen that includes the interaction of $ploidy$ and gen is

$$y = \beta_0 + \beta_1\, gen + \beta_2\, ploidy + \beta_3\, ploidy\, \times gen + \epsilon, \qquad \ldots \quad (1)$$

where ϵ is the idiosyncratic error and $ploidy\, \times gen$ models the interaction between the ploidy and generation. We fit this model separately for the fields and years using the averaged phenotypic trait values over biological replicates of a genotype.

To answer the first question in a given year, we perform the following hypothesis test

$$H_0\colon \beta_3 = 0 \quad \text{vs} \quad H_1\colon \beta_3 \neq 0$$

separately for all the genotypes and phenotypes using the regression model in (1) and assuming that ϵ is normally distributed with mean 0. For a given genotype, the p-values of the hypothesis test for all the phenotypes are collected, corrected for multiple comparisons using the FDR procedure, and tested at a significance level of 95%. If we reject the null hypothesis at 95% significance level for a particular phenotype and genotype, then the data provides evidence of interaction between the ploidy and generation for that phenotype and genotype combination; otherwise, there is absence of any evidence of interaction.

2. Is the inbreeding depression rate different between lines with the different genetic constitutions but the same ploidy?

There are thirteen and three types of genetic constitutions in the diploid and tetraploid plants, respectively. First, consider the fourteen genotypes in the diploid plants: "A188/B73", "A188/Oh43", "A188/W22", "B73/A188", "B73/Oh43", "B73/W22", "B73/W22/A188/Oh43", "Oh43/A188", "Oh43/A188/W22/B73", "Oh43/B73", "Oh43/W22", "W22/A188", "W22/B73", "W22/Oh43." Using "A188/B73" genotype as the baseline, we create 13 dummy variables, $geno_1, geno_2, \ldots, geno_{12}, geno_{13}$, that indicate the presence or absence of the remaining 13 genotypes. Following (1), we now fit a regression model that has the interaction terms for genotypes and generation for the diploid plants as

$$y = \beta_0 + \beta_1\, gen + \beta_{2,1}\, geno_1 + \cdots + \beta_{2,13}\, geno_{13} + \beta_{3,1}\, geno_1 \times gen + \cdots + \beta_{3,13}\, geno_{13} \times gen + \epsilon, \quad \ldots \quad (2)$$

where ϵ is the idiosyncratic error, gen denotes generation, and $geno_1, \ldots, geno_{13}$ represent the 13 genotype dummy variables with A188/B73 as the baseline category for diploid plants.

Second, for the tetraploid plants, we fit a model that is similar to (2) but has three genotypes "Oh43/A188/W22/B73", "A188/Oh43", and "W22/B73". Let $geno_1, geno_2$ be the two dummy variables indicating the presence of Oh43/A188/W22/B73 and W22/B73 genotypes, respectively, with “A188/Oh43” as the baseline. Then, we fit the following regression model for tetraploid plants:

$$y = \beta_0 + \beta_1\, gen + \beta_{2,1}\, geno_1 + \beta_{2,2} geno_2 + \beta_3\, geno_1 \times gen + \beta_{32} geno_2 \times gen + \epsilon, \quad \ldots \quad (3)$$

where ϵ, gen have the same meaning as in (2).

We answer the second question for a given ploidy, year, and phenotype. We fit the regression models in (2) and (3) and perform the following hypothesis tests for the diploid plants

$$H_0: \beta_{3,1} = \beta_{3,2} = \cdots = \beta_{3,12} = \beta_{3,13} = 0 \quad vs$$
$$H_1: At\ least\ one\ of\ these\ statements\ is\ false,$$

and

$$H_0: \beta_{3,1} = \beta_{3,2} = 0 \quad vs \quad H_1: At\ least\ one\ of\ these\ two\ statements\ is\ false$$

for the tetraploid plants. For a given ploidy, year, and trait, the p-values of the hypothesis test for all the genotype levels are collected, corrected for multiple comparisons using the FDR procedure, and tested at a significance level of 95%. If we reject the null hypothesis at 95% significance level for a particular phenotype and ploidy, then the data provides evidence of interaction between that genotype and generation for that phenotype and ploidy.

3. Does inbreeding depression occur in all the measured phenotypes?

Our interpretation of this question is as follows: does inbreeding depression occurs in all phenotype *irrespective of their* ploidy, genotype, and year? We fit the following regression

model separately for all the phenotypes

$$y = \beta_0 + \beta_1\ gen + \epsilon, \dots\ \ (4)$$

The answer to the third question is provided by the following hypothesis test

$$H_0: \beta_1 \geq 0\ \ vs\ \ H_1: \beta_1 < 0.$$

The p-values of the hypothesis test for all the phenotypes are collected, corrected for multiple comparisons using the FDR procedure, and tested at a significance level of 95%. If we reject the null hypothesis at 95% significance level, then there is evidence of inbreeding depression.

4. Is there inbreeding depression in every diploid and tetraploid genotype?

The answer to this question is a special case of the previous question. We fit the same model as in Question 3 but separately for every genotype and ploidy.

5. How is the inbreeding depression rate affected by ploidy, genetic constitution, and the interaction between ploidy and genetic constitution?

The answer to this question is based those for Questions 1 and 2. In particular, we choose the three genotypes from Question 1 that have diploid and tetraploid plants. Similar to Eq. (1), (2), and (3), we fit a regression model that has terms for modeling the interactions of ploidy and generation, polidy and genotype, and generation and genotype as follows

$$y = \beta_0 + \beta_1\ gen + \beta_{2,1}\ geno_1 + \beta_{2,2}\ geno_2 + \beta_{3,1}\ geno_1\ \times gen + \beta_{3,2}\ geno_2\ \times gen + \beta_4 ploidy + \beta_5\ ploidy\ \times gen + \beta_{6,1}\ geno_1\ \times ploidy + \beta_{6,2}\ geno_2\ \times ploidy + \epsilon,\ \ \dots\ \ (5)$$

where ϵ is the idiosyncratic error, gen denotes generation, $ploidy$ is 1 for tetraploid plants, and $geno_1, geno_2$ are the two dummy variables indicating the presence of Oh43/A188/W22/B73 and W22/B73 genotypes with A188/Oh43 as the baseline.

The hypotheses tests to answer this question are also similar to those in Questions 1 and 2. Specifically, we perform the following three hypotheses tests to test the interaction of ploidies and genotype

$$H_0: \beta_{6,1} = \beta_{6,2} = 0 \;\; vs \;\; H_1: At\ least\ one\ of\ these\ two\ statements\ is\ false,$$

the effect of genotype on inbreeding depression

$$H_0: \beta_{2,1} = \beta_{2,2} = \beta_{3,1} = \beta_{3,2} = \beta_{6,1} = \beta_{6,2} = 0 \;\; vs \;\; H_1: At\ least\ one\ of\ these\ statements\ is\ false,$$

and the effect of ploidy on inbreeding depression

$$H_0: \beta_4 = \beta_5 = \beta_{6,1} = \beta_{6,2} = 0 \;\; vs \;\; H_1: At\ least\ one\ of\ these\ statements\ is\ false.$$

Again, we collect p-values for all the phenotypes in a given year, correct the p-values for multiple comparisons using the FDR procedure, and do hypothesis tests simultaneously for all the phenotypes at a significant of 95% using the FDR-corrected p-values.

6. Are there any parental effects on inbreeding depression rate?

We will assume this question is concerned with the differences between the phenotypic values for the F1 generation across different ploidies, genotypes, and phenotypes. The question is answered by the following hypothesis test

$H_0: \beta_0 = 0 \; vs \; H_1: \beta_0 \neq 0$ in the regression model $y = \beta_0 + \beta_1 gen + \epsilon$

for a given phenotype because F1 corresponds to $gen = 0$. This is the same model as used in Question 3. Following our previous approach, we collect the p-values for all the phenotypic traits, correct them using the FDR procedure, and test hypotheses for all the phenotypes simultaneously at a significance of 95%.

7. Are the S7 lines different from their corresponding progenitor inbred lines?

We have coded the generations as 0, 1, 3, 5, and 7 and treated them as numeric variables in our regression models. We answer this question separately for the two ploidies using the regression model (1). Let $g = 0, 1, 3,5$ denote the non-S7 generations. Then, for a given g, the difference

between mean inbreeding depression rates for the diploid and tetraploid plants in a given year are

$$\Delta\mu_{2n} = \beta_2(7-g), \quad \Delta\mu_{4n} = (\beta_2+\beta_3)(7-g), \quad g = 0,1,3,5, \ ,$$

respectively. The difference of the mean inbreeding depression rates of the non-S7 generations with respect to the S7 generation plants is tested using the following two contrasts for the diploid and tetraploid plants

$$H_0: 7\beta_2 = 6\beta_2 = 4\beta_2 = 2\beta_2 = 0 \ \ vs \ \ H_1: At\ least\ one\ of\ this\ these\ statements\ is\ false$$

and

$$H_0: 7\beta_2 = 6\beta_2 = 4\beta_2 = 2\beta_2 = 0\ vs\ H_1: At\ least\ one\ of\ this\ these\ statements\ is\ false,$$

respectively. Testing these two contrasts is equivalent to testing the following simpler hypotheses

$$H_0: \beta_2 = 0\ vs\ H_1: \beta_2 \neq 0$$

and

$$H_0: \beta_2 + \beta_3 = 0\ vs\ H_1: \beta_2 + \ \beta_3 \neq 0$$

for the diploid and tetraploid, respectively. We do these two hypotheses tests separately for all the phenotypes, collect p-values, and test hypotheses after correcting for multiple comparisons using the FDR procedure.

3. Results

The summary of the results for all the seven questions discussed in the previous sections are as follows:

1. Is the inbreeding depression rate different between diploid and tetraploid lines with the same genetic constitution?

The data provide *no evidence* of an interaction between ploidy and generation in all the three genotypes at a 95% level of statistical significance for the two fields in 2008 data and for the second and third fields in the 2009 data. The only exception is field 1 in 2009 data that in which

Tassel Branch phenotype is significant.

The FDR-corrected p-values of the hypothesis tests are stored as csv files under the name *genotype_coefs_field_1/2/3*, where genotype is any of the three genotypes (Supplement 4).

2. Is the inbreeding depression rate different between lines with the different genetic constitutions but the same ploidy?

The data provide *no evidence* of an interaction between genetic constitution and generation in both ploidies at a 95% level of statistical significance for the two fields in 2008 data and for the three fields in the 2009 data with some exceptions. Specifically, in the 2009 data and field 1,

- the interaction of A188/Oh43 and 2x ploidy is significant for Tassel Branch phenotype;
- the interaction of W22/B73 and 4x ploidy is significant for Flower Time and Silk Time phenotypes.

In field 2, only the interaction of 4x ploidy and W22/B73 genotype is significant for the fifth leaf length phenotype.

The FDR-corrected p-values of the hypothesis tests are stored as csv files under the name *2n/4n_coefs_field_1/2/3* (Supplement 4).

3. Does inbreeding depression occur in all the measured phenotypes?

The answer is yes for the two fields in 2008 data and for the three fields in the 2009 data, excluding the Flower Time and Silk Emergence Time phenotypes.

The FDR-corrected p-values of the hypothesis tests are stored as csv files under the name *coef_field_1/2/3* (Supplement 4).

4. Is there inbreeding depression in every diploid and tetraploid genotype?

The FDR-corrected p-values of the hypothesis tests are stored as csv files under the name

genotype_phenotype_coef_2n/4n_field_*1/2/3* for the diploid and tetraploid plants. These files have been included as separate files with the significant phenotype and genotype combinations in csv files with a "*sig*" appended to their names (Supplement 4). Below is a summary of the significant genotypes and phenotypes depending on the year, field, and ploidy.

2008 Data

Field 2

2x ploidy

Genotype	Phenotype
A188/Oh43	Ear_Length
A188/W22	5th_Leaf_Length
A188/W22	7th_Leaf_Width
Oh43/A188	Height_at_4_Week
Oh43/A188	Adult_Plant_Height
Oh43/A188	5th_Leaf_Length
Oh43/A188	7th_Leaf_Length
Oh43/A188	7th_Leaf_Width
Oh43/B73	5th_Leaf_Length
Oh43/B73	5th_Leaf_Width
Oh43/W22	Tassel_Branch
W22/A188	5th_Leaf_Length
W22/Oh43	Height_at_4_Week
W22/Oh43	Adult_Plant_Height
W22/Oh43	Tassel_Branch
W22/Oh43	5th_Leaf_Width

4x ploidy

Genotype	Phenotype
A188/Oh43	7th_Leaf_Length
Oh43/A188/W22/B73	Ear_Length

Oh43/A188/W22/B73	7th_Leaf_Length
W22/B73	Ear_Length
W22/B73	7th_Leaf_Width

Field 3

2x ploidy

Genotype	Phenotype
B73/Oh43	Tassel_Branch
B73/W22	7th_Leaf_Length
Oh43/W22	Tassel_Branch

4x ploidy

Genotype	Phenotype
A188/Oh43	Ear_Length
A188/Oh43	Adult_Plant_Height
A188/Oh43	5th_Leaf_Length
A188/Oh43	7th_Leaf_Length
A188/Oh43	5th_Leaf_Width
Oh43/A188/W22/B73	7th_Leaf_Length
Oh43/A188/W22/B73	Tassel_Branch
W22/B73	Ear_Length
W22/B73	Height_at_4_Week

<u>2009 Data</u>

Field 1

2x Ploidy

Genotype	Phenotype
B73/A188	Height_at_4_Week
B73/W22	Height_at_4_Week
B73/W22xA188/Oh43	Height_at_4_Week

B73/W22xA188/Oh43	Adult_Plant_Height
W22/Oh43	Tassel_Branch

4x Ploidy

**Genotype**	**Phenotype**
A188/Oh43	Height_at_4_Week
A188/Oh43	Height_at_6_Week
Oh43/A188/W22/B73	Height_at_4_Week
Oh43/A188/W22/B73	5th_Leaf_Length
W22/B73	Ear_Length

Field 2

2x Ploidy

None

4x Ploidy

**Genotype**	**Phenotype**
A188/Oh43	Adult_Plant_Height
A188/Oh43	5th_Leaf_Width
A188/Oh43	Ear_Length
Oh43/A188/W22/B73	Height_at_4_Week
Oh43/A188/W22/B73	Height_at_6_Week
Oh43/A188/W22/B73	Tassel_Branch
Oh43/A188/W22/B73	Ear_Length
W22/B73	Tassel_Branch
W22/B73	Ear_Length

Field 3

2x Ploidy

None

4x Ploidy

**Genotype**	**Phenotype**
A188/Oh43	Height_at_4_Week
A188/Oh43	Height_at_6_Week
A188/Oh43	5th_Leaf_Width
A188/Oh43	Tassel_Branch
A188/Oh43	Ear_Length
Oh43/A188/W22/B73	Height_at_4_Week
Oh43/A188/W22/B73	Height_at_6_Week
Oh43/A188/W22/B73	5th_Leaf_Width
Oh43/A188/W22/B73	Tassel_Branch
Oh43/A188/W22/B73	Ear_Length

5. How is the inbreeding depression rate affected by ploidy, genetic constitution, and the interaction between ploidy and genetic constitution?

There are two interactions between ploidy and genotype that are significant in the 2008 data: *ploidy and W22/B73 genotype* for the *fifth leaf length trait* in *field 2* and *ploidy and A188/Oh43 genotype* for the *seventh leaf width* phenotype in field 3. The number of significant interactions in the 2009 data are much larger and are summarized below:

Field 1

Ploidy and Genotype

**A188/Oh43**	**W22/B73**
Tassel_Branch	Tassel_Branch
None	Height_at_6_Week

Field 2

Ploidy and Genotype

A188/Oh43	W22/B73
None	Height_at_4_Week
None	Height_at_6_Week

Field 3

Ploidy and Genotype

A188/Oh43	W22/B73
0.99527163	Height_at_4_Week
Tassel_Branch	Tassel_Branch
None	Height_at_6_Week
5th_Leaf_Width	None

The FDR-corrected p-values of the hypothesis tests are stored as csv files under the names *ploidy_genotype_int_pval_fdr_corrected_field_1/2/3*, *genotype_pval_fdr_corrected_field_1/2/3*, and *ploidy_ int_pval_fdr_corrected_field_1/2/3*, respectively (Supplement 4).

6. Are there any parental effects on inbreeding depression rate?

F0 is significant for every phenotype and field in 2008 and 2009 data. The FDR-corrected p-values of the hypothesis tests are stored as csv files under the name *coef_field_1/2/3* (Supplement 4).

7. Are the S7 lines different from their corresponding inbred lines?

The FDR-corrected p-values of the hypothesis tests are stored as csv files under the name *genotype_S7vsRest_field_1/2/3*, where genotype is any of the three genotypes (Supplement 4). Below is a summary of the significant genotypes and phenotypes depending on the year, field, and ploidy.

2008 Data

A188/Oh43

Field 2

None

Field 3

**Diploid**	**Tetraploid**
Flower_Time	Flower_Time
Silk_Time	Silk_Time
5th_Leaf_Width	5th_Leaf_Width
Height_at_4_Week	None
5th_Leaf_Length	5th_Leaf_Length
7th_Leaf_Width	7th_Leaf_Width
Ear_Length	Ear_Length
7th_Leaf_Length	7th_Leaf_Length
None	Adult_Plant_Height

Oh43/A188/W22/B73

Field 2

Diploid	Tetraploid
Ear_Length	Ear_Length

Field 3

**Diploid**	**Tetraploid**
7th_Leaf_Width	None
Silk_Time	None
5th_Leaf_Width	None

W22/B73

Field 2

None

Field 3

**Diploid**	**Tetraploid**
None	Ear_Length

2009 Data

A188/Oh43

Field 1

**Diploid**	**Tetraploid**
None	Height_at_4_Week
Height_at_6_Week	Height_at_6_Week
Flower_Time	Flower_Time
Silk_Time	Silk_Time
None	5th_Leaf_Width

Field 2

**Diploid**	**Tetraploid**
Flower_Time	Flower_Time
5th_Leaf_Width	5th_Leaf_Width
Height_at_4_Week	Height_at_4_Week
Height_at_6_Week	Height_at_6_Week
Silk_Time	Silk_Time
None	Ear_Length

Field 3

**Diploid**	**Tetraploid**
Height_at_4_Week	Height_at_4_Week

None	5th_Leaf_Width
None	Tassel_Branch
Height_at_6_Week	Height_at_6_Week
Flower_Time	Flower_Time
Silk_Time	Silk_Time
None	Ear_Length

Oh43/A188/W22/B73

Field 1

None

Field 2

**Diploid**	**Tetraploid**
Height_at_4_Week	Height_at_4_Week
Height_at_6_Week	Height_at_6_Week
Flower_Time	None
Silk_Time	None
5th_Leaf_Width	None
Adult_Plant_Height	None

Field 3

**Diploid**	**Tetraploid**
Height_at_6_Week	Height_at_6_Week
Height_at_4_Week	Height_at_4_Week
None	Tassel_Branch
Ear_Length	Ear_Length
Flower_Time	Flower_Time
Silk_Time	Silk_Time

W22/B73

Field 1

**Diploid**	**Tetraploid**
Adult_Plant_Height	None

Field 2

None

Field 3

None

4. Output Files

All the .csv files containing results of the analyses are included as separate files (Supplement 4). The relevant .csv files summarizing results of *Questions 1* to *7* for 2008 and 2009 data are separately assembled, respectively.

Appendix

The R code and results for the two questions are included in supplementary files (Supplement 4). Each file contains *code*, *data*, and *result* folders. The results are in the *result* folder and are summarized in subfolders *q1, q2, q3, q4, q5, q6, q7*.

References

Faraway, J. J., 2006 Extending the Linear Model with R. Chapman & Hall, Boca Raton.
